# Hydrolysis of Acetamide on Low-Index CeO_2_ Surfaces: Ceria as a Deamidation and General De-esterification Catalyst

**DOI:** 10.1021/acscatal.2c02514

**Published:** 2022-08-05

**Authors:** Suman Bhasker-Ranganath, Ye Xu

**Affiliations:** Cain Department of Chemical Engineering, Louisiana State University, Baton Rouge, Louisiana 70803, United States

**Keywords:** deamidation, de-esterification, hydrolysis, generalized ester, enzyme mimic, ceria, density functional theory, descriptor

## Abstract

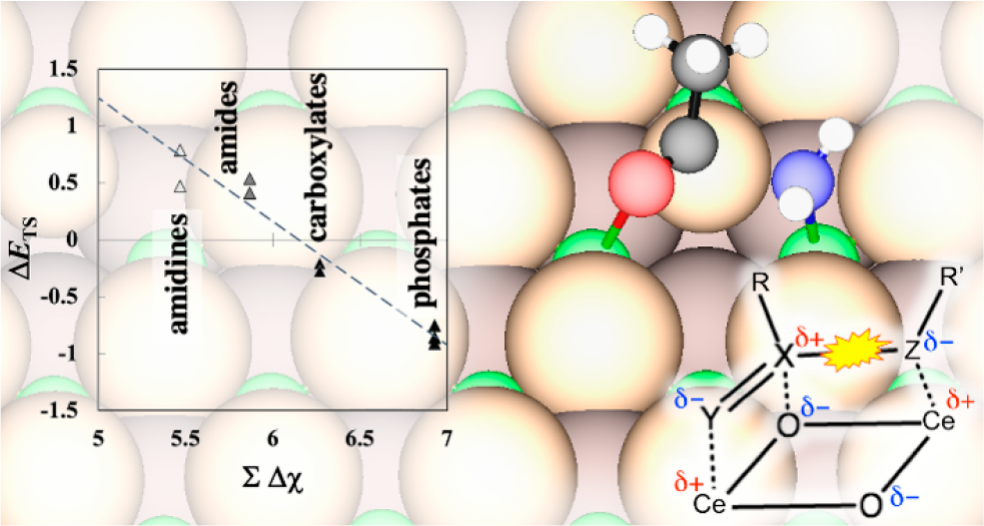

Using DFT calculations and acetamide as the main example, we show that ceria is a potential catalyst for the hydrolysis of amide and similar bonds. The overall reaction is endergonic in the gas phase, yielding acetic acid and ammonia, but is slightly exergonic in the aqueous phase, which facilitates ionization of the products (CH_3_COO^–^ and NH_4_^+^). Neighboring Ce and O sites on the CeO_2_(111), (110), and (100) facets are conducive to the formation of an activated metastable tetrahedral intermediate (TI) complex, followed by C–N bond scission. With van der Waals and solvation effects taken into account, the overall reaction energetics is found to be most favorable on the (111) facet as desorption of acetic acid is much more uphill energetically on (110) and (100). We further suggest that the Ce–O–Ce sites on ceria surfaces can activate X(=Y)–Z type bonds in amides, amidines, and carboxylate and phosphate esters, among many others that we term “generalized esters”. A Brønsted-Evans–Polanyi relationship is identified correlating the stability of the transition and final states of the X–Z generalized ester bond scission. A simple descriptor (ΣΔχ) based on the electronegativity of the atoms that constitute the bond (X, Y, Z) versus those of the catalytic site (O, Ce, Ce) captures the trend in the stability of the transition state of generalized ester bond scission and suggests a direction for modifying ceria for targeting specific organic substrates.

## 1 Introduction

The stability of peptides, proteins, antibodies, and some polymers is largely attributed to their amide (C(=O)–N) bonds, which retain a partial double bond character via resonance stabilization.^1^ Large rotational barriers associated with the amide C–N bonds help preserve the 3D structure of amino acid chains in proteins.^2^ Scission of peptide bonds in peptides and proteins (deamidation), and of C–N bonds in related C(=X)–N compounds, such as amidines, plays a fundamental role in proteomics, therapeutics, chemical genetics, analytical biochemistry, and biotechnology. In environmental and biochemical settings, the reaction proceeds through the formation of a tetrahedral intermediate (TI) following nucleophilic attack at the carbonyl C by water.^3,4^ A base-catalyzed mechanism, in which a hydroxide group functions as the nucleophile, appears to be operative even in neutral aqueous solutions.^5−7^ Oxygen exchange between amides and water is observed, which is attributed to reversible formation of the TI. The dissociation of the C–N bond in the TI is the rate-limiting step, in contrast to the dissociation of the C–O bond in the hydrolysis of carboxylate esters where the formation of the TI is rate-limiting because alkoxides (−OR) are better leaving groups than amides (−NR_2_).^4,8^ At neutral pH and ambient temperature, peptide bonds have a long half-life of 250–600 years, with respect to nonenzymatic hydrolysis.^9,10^

Enzymes such as peptidase and deaminase catalyze the hydrolysis of amide C–N bonds in a narrow range of pH and temperature. Many peptidases do not contain metals, while a particular subclass, metallopeptidase, does use metal cations to catalyze the reaction. Representative members of this class include carboxypeptidase A and thermolysin, both of which contain a Zn^2+^ at their active sites. How Zn^2+^ accelerates amide C–N bond scission has long been debated.^11−13^ The role of Zn^2+^ is currently understood as positioning the carbonyl group by coordinating to its O atom, stabilizing a water molecule or hydroxide group that attacks the carbonyl C, and stabilizing one or both negatively charged C–O groups.^13,14^ There is also evidence that intramolecular nucleophilic attack by an hydroxyl or thiol group that is a part of certain amino acids (e.g., serine, threonine, and cysteine) on the central C helps break the amide C–N bond under unfavorable external pH conditions, which is referred to as N–O/S acyl rearrangement.^15,16^

Interest in artificial metallopeptidase has been growing.^14,17,18^ Based on the understanding of the action of metallopeptidase, complexes of Cu^2+^,^19,20^ Pd^2+^,^21^ Pt^2+^,^22^ and Co^3+^ (ref (23)) have been investigated for catalytic hydrolysis of amides and peptides under mild conditions. Kita et al. reported Zn acetate and Zn triflate to have higher deamidation activity than Co, Mn, and Cu acetates, while Pd, Ni, and Ag acetates have no activity.^24^ Even the inorganic salt ZnCl_2_ has noticeable deamidation activity, but Zn dust, which is likely oxidized Zn, has little activity.^24,25^ Alcohols can be used in lieu of water for the alcoholysis of amides.^24^

Solid heterogeneous catalysts have certain advantages over homogeneous catalysts with respect to recovery and separation from reaction media. Since the first reports in 1950s,^26,27^ complexes of Ce^4+^ have been investigated for catalyzing the hydrolysis of small peptides^28,29^ and proteins^30−33^ in homogeneous solutions. Higher conversions were reported for Ce^4+^ complexes than Ce^3+^ and other metal ion species, which is attributed to the large coordination number of Ce^4+^ and its ability to form a more stable reduced Ce^3+^ state by withdrawing electrons from the amide linkage. This has led to investigation into solid ceria (CeO_2_) as a possible deamidation catalyst. Over the past decade, Shimizu and co-workers demonstrated efficient alcoholysis and subsequent esterification of amides over high loadings of CeO_2_ in batch mode in hot, boiling alcohols.^34−37^ Recently, the same group investigated the hydrolysis of acetamide under similar conditions of boiling solvents and high catalyst loadings using various solid oxides including Nb_2_O_5_, ZrO_2_, CeO_2_, TiO_2_, SiO_2_, and Al_2_O_3_, among others as catalysts.^38^ The first three oxides were reported to achieve conversion of acetamide to acetic acid over 45%, including 100% conversion over Nb_2_O_5_. The authors proposed mechanisms for the hydrolysis and alcoholysis of amides based on the concerted actions of Lewis-acidic metal cation and basic O anion that attack the carbonyl O and carbonyl C, respectively, and supported them with density functional theory (DFT) calculations on CeO_2_(111).^35,38^ They also identified a correlation between the increasing basicity of lattice oxygen in metal oxides, as represented by the O_1s_ binding energy, and higher initial reaction rates for several oxides. It remains unclear why CeO_2_ is more effective than Nb_2_O_5_ in alcoholysis^37^ but not in hydrolysis.^38^

Ceria-catalyzed hydrolysis and alcoholysis reported thus far in the literature have been based on commercially available polycrystalline ceria powder.^34−38^ It is not clear which low-index facet or what surface structure, if any, plays a dominant role in the observed deamidation activity. Surface structure sensitivity has been observed for many reactions on ceria.^39−42^ Mullins and co-workers showed that small organic molecules, including water, alcohols, acetic acid, and acetaldehyde, exhibit qualitatively different temperature-programmed desorption (TPD) results on CeO_2_(111) vs CeO_2_(100) thin films.^43^ Ceria nanocubes and nanorods, which predominantly expose (100) and/or (110) facets, and nano-octahedra, which predominantly expose (111) facets, exhibit different activities or product distributions when catalyzing a wide range of reactions including CO oxidation,^44^ ethanol oxidation,^45^ carbamoylation,^46^ CO_2_–methanol coupling,^47^ and aqueous-phase dephosphorylation.^48^ The differences are variously attributed to factors including different binding strength, basicity of lattice oxygen, and reducibility of the facets.^49,50^

**Figure 1:**
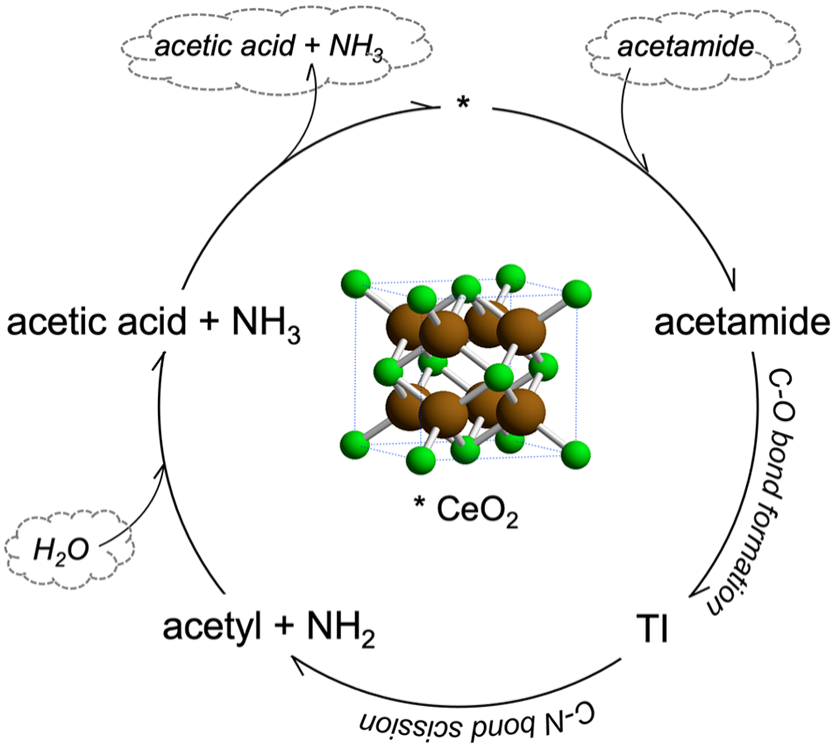
Schematic illustrating the overall reaction pathway for acetamide deamidation and hydrolysis on ceria. Asterisk symbol (*) denotes catalytic sites. TI stands for tetrahedral intermediate. Species in cloud are located off the catalyst.

In the present work, we perform periodic DFT calculations to examine in detail the mechanism of hydrolysis of the amide C–N bond, using acetamide as the main model compound, on the three low-index facets of CeO_2_: (111), (110), and (100). The basic reaction pathway, as will be presented and discussed below, is outlined in Figure 1. Because of the significance of deamidation reactions in biochemical contexts, the effects of solvation and dispersion interactions are taken into consideration using an implicit solvation model and a van der Waals functional, respectively. We further consider C–N bond scission in benzamide, *N*-methylacetamide, acetamidine, and adenine on CeO_2_(111) to highlight the ability of the Ce–O–Ce site ensemble to adsorb and activate C(=X)–N bonds, which is representative of the reactivity of ceria toward an even broader class of heteroatomic organic compounds containing the motif of X(=Y)–Z, including organic amides, phosphates, and carboxylates, all of which we term generalized esters (GEs). Our results shed light on the fundamental properties that endow ceria with versatile reactivity toward biologically and environmentally relevant organic molecules, including peptides and nucleobases. They make ceria a promising enzyme-mimetic material for the development of novel diagnostic, pharmaceutical, and therapeutic technologies, among others.^51−53^ The main challenge for ambient temperature hydrolysis applications is identified to be the desorption of the carboxylate product.

## 2 Methods

Periodic DFT calculations were performed within the generalized gradient approximation (GGA-PW91^54^) and with van der Waals corrections (optB86b-vdW),^55−58^ using the Vienna Ab initio Simulation Package (VASP)^59−62^ and Quantum Espresso (QE).^63,64^ The potentials due to core electrons were described using the projector-augmented wave (PAW) method,^65,66^ and the Kohn–Sham valence states were expanded in a plane wave basis set with a cutoff energy of 400 eV in VASP. PAW potentials for Ce, O, and C, and ultrasoft pseudopotentials (USPP) for H and N, all taken from the standard solid-state pseudopotentials (SSSP) library v1.1.2,^67^ were used in QE.

**Figure 2:**
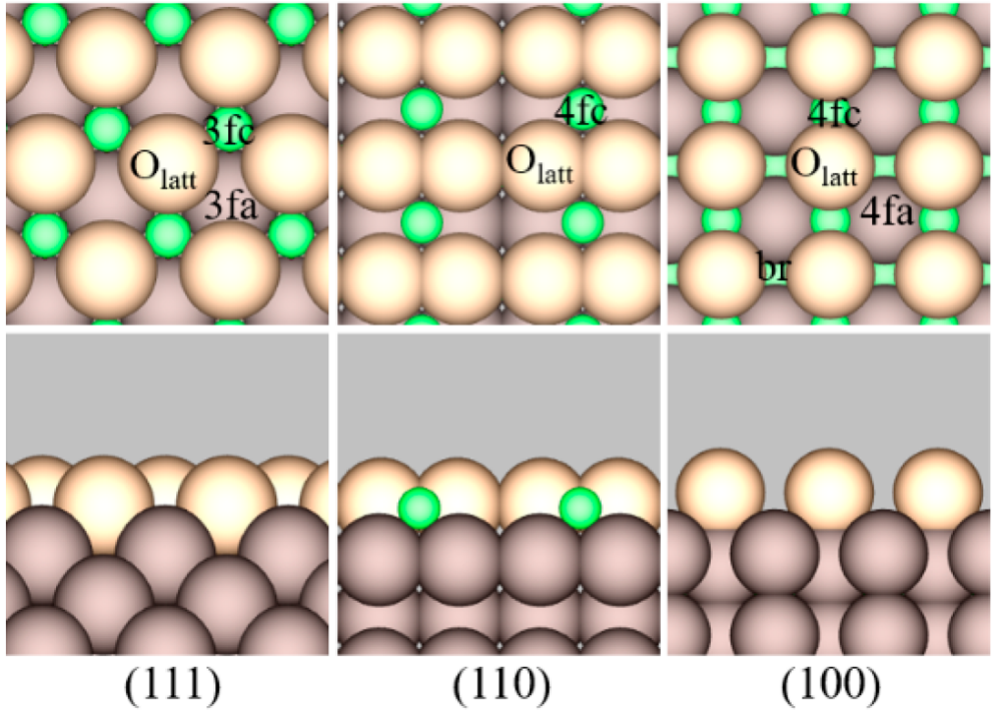
Top (top panels) and side (bottom panels) views of the respective low-index stoichiometric CeO_2_ facets, with the surface sites labeled. [Color code: green, Ce; light brown, surface O_latt_; and dark brown, subsurface O_latt_. Site designations: 3fa, 3fc, 4fa, 4fc, and br refer to the 3-fold anion, 3-fold cation, 4-fold anion, 4-fold cation, and bridge sites, respectively.]

The (111), (110), and (100) facets of CeO_2_ were modeled as p(3 × 3) slabs (Figure 2) of 9, 4, and 9 atomic layers (corresponding to 3, 4, and 4 O–Ce–O trilayers), with periodic images of the slabs separated by 12–16 Å of vacuum and dipole decoupling applied in the *z*-direction. A Γ-centered Monkhorst–Pack 2 × 2 × 1 *k*-point grid was used to sample the surface Brillouin zone.^68^ Adsorption of a single molecule per surface unit cell resulted in 1/9 monolayer (ML) coverage. The top three, two, and five atomic layers of the (111), (110), and (100) slabs were relaxed, respectively, while the remainder of each slab was held fixed at bulk coordinates. Oxygen vacancies were not considered, because those in the surface and near-surface regions are readily blocked or annihilated by the oxygenate species involved in this reaction system such as water and acetate,^69−71^ and more generally, by O_2_ and water occupying and dissociating in them in ambient settings. Geometry optimization was performed until the maximum residual force was 0.03 eV/Å or less in each relaxed degree of freedom.

The adsorption energy (Δ*E*_ads_) was evaluated as Δ*E*_ads_ = *E*_total_ – *E*_slab_ – *E*_gas_, where *E*_total_, *E*_slab_, and *E*_gas_ refer to the total energies of a slab with an adsorbate, the slab without any adsorbate, and the isolated adsorbate in gas phase, respectively. A more negative value of Δ*E*_ads_ indicates stronger bonding between an adsorbate and a slab. Gas-phase species were optimized in a (15 Å)^3^ simulation cell, with dipole decoupling applied in all directions in VASP^72^ and with the Martyna–Tuckerman correction applied in QE.^73^

The elementary steps with associated transition states (TS) in the catalytic C–N bond scission and hydrolysis of acetamide and other GEs were determined using the climbing-image nudged elastic band method^74,75^ and dimer method^76,77^ until the maximum residual force converged to 0.03 eV/Å or less in all relaxed degrees of freedom. The reaction energy (Δ*E*_rxn_) was evaluated as Δ*E*_rxn_ = *E*_FS_ – *E*_IS_, and the activation barrier (*E*_a_) was evaluated as *E*_a_ = *E*_TS_ – *E*_IS_, where *E*_IS_, *E*_TS_, and *E*_FS_ are the total energies of the initial, transition, and final states, respectively. Each TS was verified to possess only one vibrational mode with a negative curvature in the direction of the reaction under consideration. Vibrational modes and frequencies were calculated using finite difference approximation of the dynamical matrix with a displacement of 0.01 Å. Infrared spectra of adsorbed states were simulated using Atomic Simulation Environment (ASE).^78^

The DFT+U approach of Dudarev et al.^79^ was employed to partially rectify the delocalization of Ce 4f states resulting from self-interaction error.^50^ A *U*_eff_ value of 2 eV was used in this study based on better agreements between GGA+U predictions obtained at *U*_eff_ = 2–3 eV and available experimental measurements, including the bulk reduction energy of CeO_2_,^80,81^ chemisorption energy of CO on CeO_2_(110),^82^ and peak desorption temperatures of AcH.^83^ The calculated equilibrium lattice constants of 5.476 Å (GGA-PW91) and 5.452 Å (optB86b-vdW)^84^ are in good agreement with the experimental value of 5.41 Å.^85,86^

The effects of solvation by water were modeled implicitly by treating the solvent as a dielectric continuum. While VASPsol^87,88^ has been popularly used to study solvation effects for a variety of molecules and extended structures, self-consistent field (SCF) cycles failed to converge for the CeO_2_ facets. Indeed, there has been no study reporting the application of VASPsol to modeling solvation of CeO_2_ surfaces, to the best of our knowledge. Therefore, VASPsol was applied only to isolated molecular species at default settings. For periodic CeO_2_ slabs, we used the self-consistent continuum solvation (SCCS) model as implemented in Environ for QE,^89,90^ with permittivity set to 78.3 and surface tension and pressure both set to 0. A higher cutoff energy of 35 Ry, or 476 eV, was used to improve SCF convergence. While the maximum residual forces converged to below 0.04 eV/Å for molecules, ions, and the clean CeO_2_ slabs, they could not be reduced to below 0.3, 0.1, and 0.1 eV/Å for some of the proposed reaction intermediates adsorbed on the (111), (110), and (100) slabs, respectively.

## 3 Results and Discussion

### 3.1 Adsorption of Acetamide

**Figure 3:**
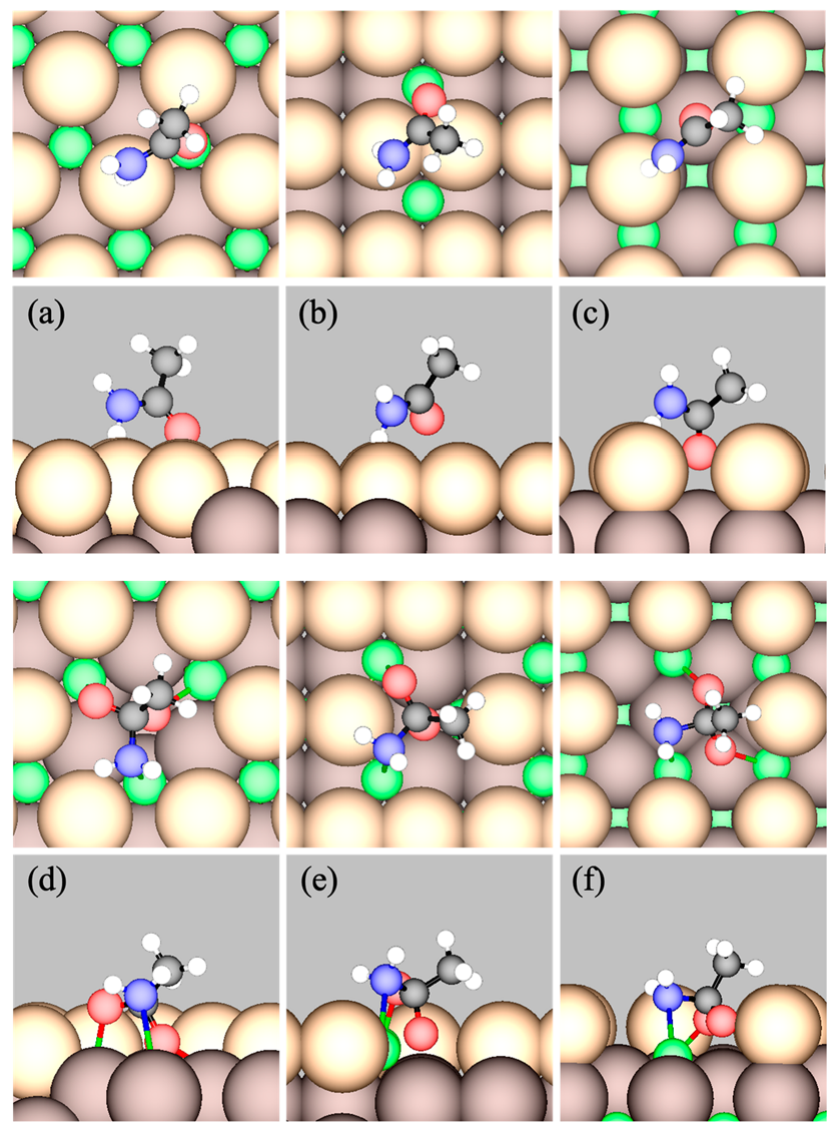
Top (top panels) and side (bottom panels) views of GGA-PW91 optimized acetamide adsorbed in the (a–c) η^1^ and (d–f) TI state on the left, (111); middle, (110); and right, (100) facets of CeO_2_ respectively. [Color code: green, Ce; light brown, surface O_latt_; dark brown, subsurface O_latt_; red, O in molecule and O_latt_ directly bonded to C; black, C; blue, N; and white, H.]

Two stable molecular adsorption configurations can be found for acetamide on the stoichiometric CeO_2_ facets: the η^1^ state (Figures 3a–c), in which acetamide is located above a 3fc or 4fc site through C=O with one of the amine H atoms simultaneously interacting with a neighboring O atom in the oxide surface (denoted O_latt_; see Figure 2); and a metastable state, in which acetamide binds to an O_latt_ through its central carbonyl C, with the carbonyl O and the amine N each coordinated to an adjacent Ce (Figures 3d–f). Strong Lewis acid–base interaction between the Ce cation and the carbonyl oxygen of acetamide is previously concluded by Kamachi et al.^35^ Key bond distances in the minimum-energy structures shown in Figure 3 are reported in Table 1.

**Table 1:** GGA-PW91 Adsorption Energy (Δ*E*_ads_) and Key Bonding Distances of Acetamide Adsorbed in the η^1^ and TI States, and Activation Barrier for Converting η^1^ into TI (*E*_a_) on the Three Low-Index Facets of CeO_2_

				Bond Distances (Å)				
state	facet	Δ*E*_ads_ (eV)	*E*_a_ (eV)	C–O_latt_	N–Ce	O–Ce	C–N	C=O
gas phase	–	–	–	–	–	–	1.37	1.23
								
η^1^	(111)	–0.64	0.65	–	–	2.56	1.34	1.25
	(110)	–0.88	0.57	–	–	2.56	1.34	1.25
	(100)	–1.36	0.80	–	–	2.53/2.54^a^	1.32	1.28
								
TI	(111)	–0.32		1.45	2.71	2.28	1.50	1.36
	(110)	–0.74		1.43	2.62	2.26	1.53	1.38
	(100)	–1.40		1.41	2.74	2.33/2.67^a^	1.52	1.40

a O–Ce bond distances are between =O and the two Ce constituting the bridge site.

Adsorption in the η^1^ state causes minimal changes in the geometry of acetamide, which appears to be a nonactivated process. The η^1^ state, because of its polarized C=O and C–N bonds, is open to nucleophilic attack on the central C by a nucleophile, e.g., O_latt_ in the surface. It must overcome an activation barrier as the sp^2^ hybridization of the >C=O group transitions to sp^3^ hybridization that is seen in the TI in the hydrolysis of amide and peptide bonds by biochemists.^4,7,11,23,91,92^ In metal ion-catalyzed hydrolysis, binding of the carbonyl O to a metal ion, followed by nucleophilic attack by water or OH, results in the formation of the TI complex. In contrast, here the TI is formed directly on CeO_2_ surfaces without an additional nucleophile due to the presence of adjacent acid–base sites. The calculated *E*_a_ values for this conversion (step b → c, Table 2) are 0.65, 0.57, and 0.80 eV on the (111), (110), and (100) facets, respectively. For comparison, Kamachi et al. calculated this *E*_a_ value to be 0.73 eV for acetamide on (111) using GGA-PBE and an atomic basis set.^35^ Similar complexes have been previously predicted theoretically for dimethylcarbonate,^46,93^ benzyl acetate,^35^ and phosphate monoesters on CeO_2_(111).^94,95^ The C=O and C–N bonds lengthen significantly from the η^1^ state to the TI state (Table 1), suggesting the latter to be a precursor state to the dissociation of the C–N bond.

The adsorption energies (Δ*E*_ads_) of the molecular species adsorbed on the three CeO_2_ facets in this reaction calculated using GGA-PW91 and optB86b-vdW are listed in Table S1 in the Supporting Information, together with available literature values. Δ*E*_ads_ consistently follows the trend (111) > (110) > (100) (i.e., adsorption most stable on (100)), according to both GGA-PW91 and optB86b-vdW, including acetamide in the η^1^ and TI states. The trend reflects the fact that the combined local coordination of the topmost Ce and O_latt_ atoms is the lowest on (100) and highest on (111).^96^ Stronger binding of the products (acetic acid and NH_3_) than the reactants is evident.

Kamachi et al. reported Fourier transform infrared (FTIR) spectra of gas-phase acetamide exposed to polycrystalline CeO_2_ at 120 °C.^35^ Three prominent peaks were found at 1430, 1554, and 1656 cm^–1^. The 1656 cm^–1^ peak was attributed to the ν(C=O) mode of η^1^ acetamide, the intensity of which decreased appreciably on the order of minutes. This assignment agrees with our simulated IR spectra for acetamide (see Figure S1 in the Supporting Information), which put this mode at 1620–1640 cm^–1^ on the three lowest-index facets of ceria. The mode is absent in the TI, nor is IR detection of it expected, because of the metastable nature of the TI and facile C–N bond scission (see the next section). The other two peaks in the FTIR increased in intensity with time and were attributed to acetate species. The peaks at 1430 and 1554 cm^–1^ agree with previous IR studies of acetic acid adsorbed on CeO_2_(111)^71^ and polycrystalline ceria.^97,98^ They are consistent with our analysis below, which suggests acetate to be desorption-limited.

### 3.2 Deamidation and Hydrolysis of Acetamide

**Figure 4:**
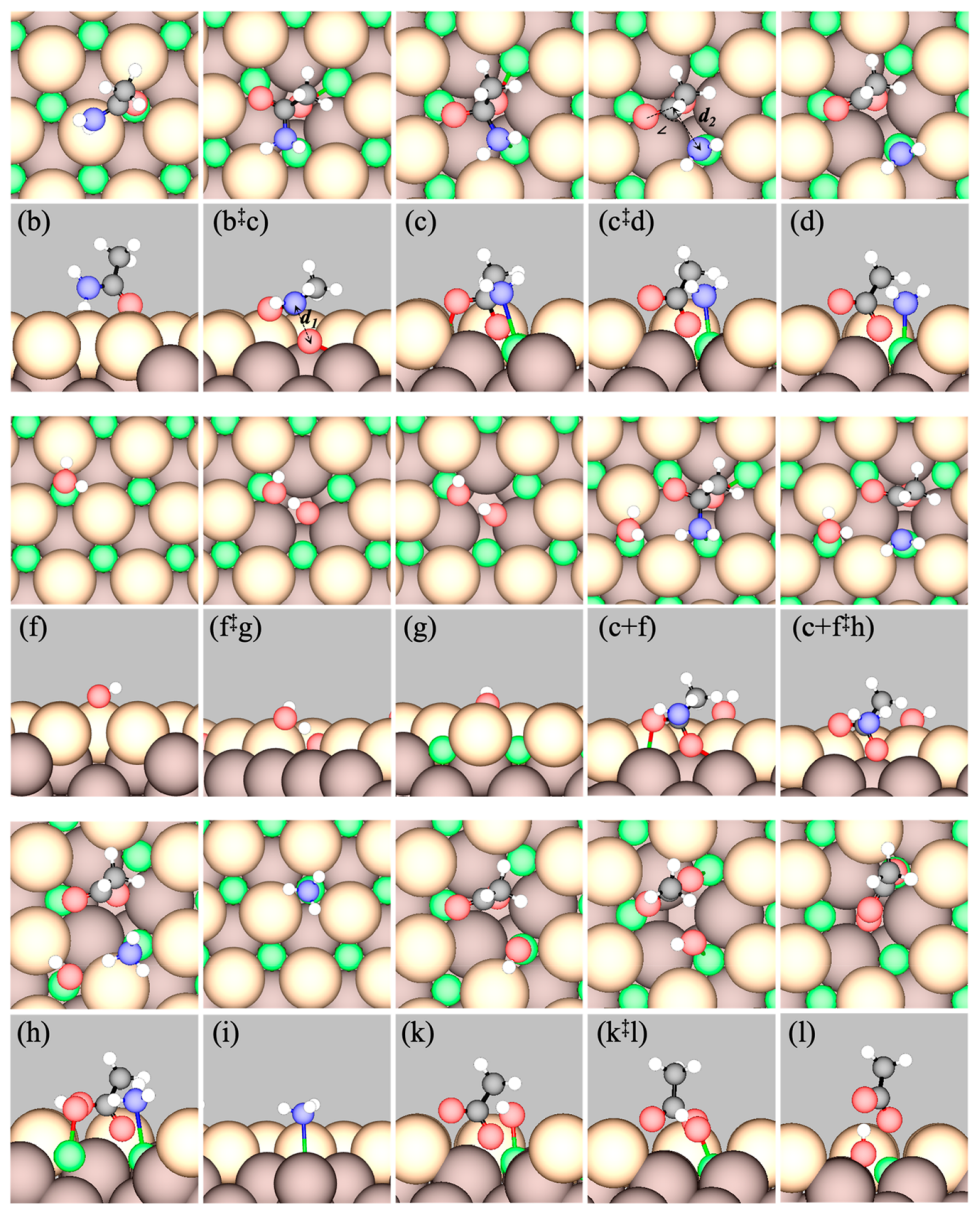
Top (upper panels) and side (lower panels) views of GGA-PW91 optimized stable intermediates and TSs (indicated by ^‡^) of the elementary steps in deamidation and hydrolysis of acetamide on stoichiometric CeO_2_(111). Labels of states correspond to those in Table 2. The states shown are (b) η^1^ acetamide, (b^‡^c) TS of nucleophilic attack by O_latt_, (c) TI, (c^‡^d) TS of C–N scission, (d) acetyl + NH_2_, (f) H_2_O, (f^‡^g) TS of H_2_O dissociation, (g) OH + H, (c + f) TI + H_2_O, (c + f^‡^h) TS of C–N scission with coadsorbed water, (h) acetyl + NH_3_ + OH, (i) NH_3_, (k) acetyl + OH, (k^‡^l) TS of OH attack, and (l) acetate + H. [Color code: green, Ce; light brown, surface O_latt_; dark brown, subsurface O_latt_; red, O in molecules; black, C; blue, N; and white, H.] O_latt_ bonded to C or H atoms in the molecules are considered part of the molecules. At b^‡^c, the length of the transitioning C–O_latt_ bond is *d*_1_ = 1.90 Å. At c^‡^d, the length of the transitioning C–N bond is *d*_2_ = 2.25 Å and the angle ∠OCN is 106.1°.

**Figure 5:**
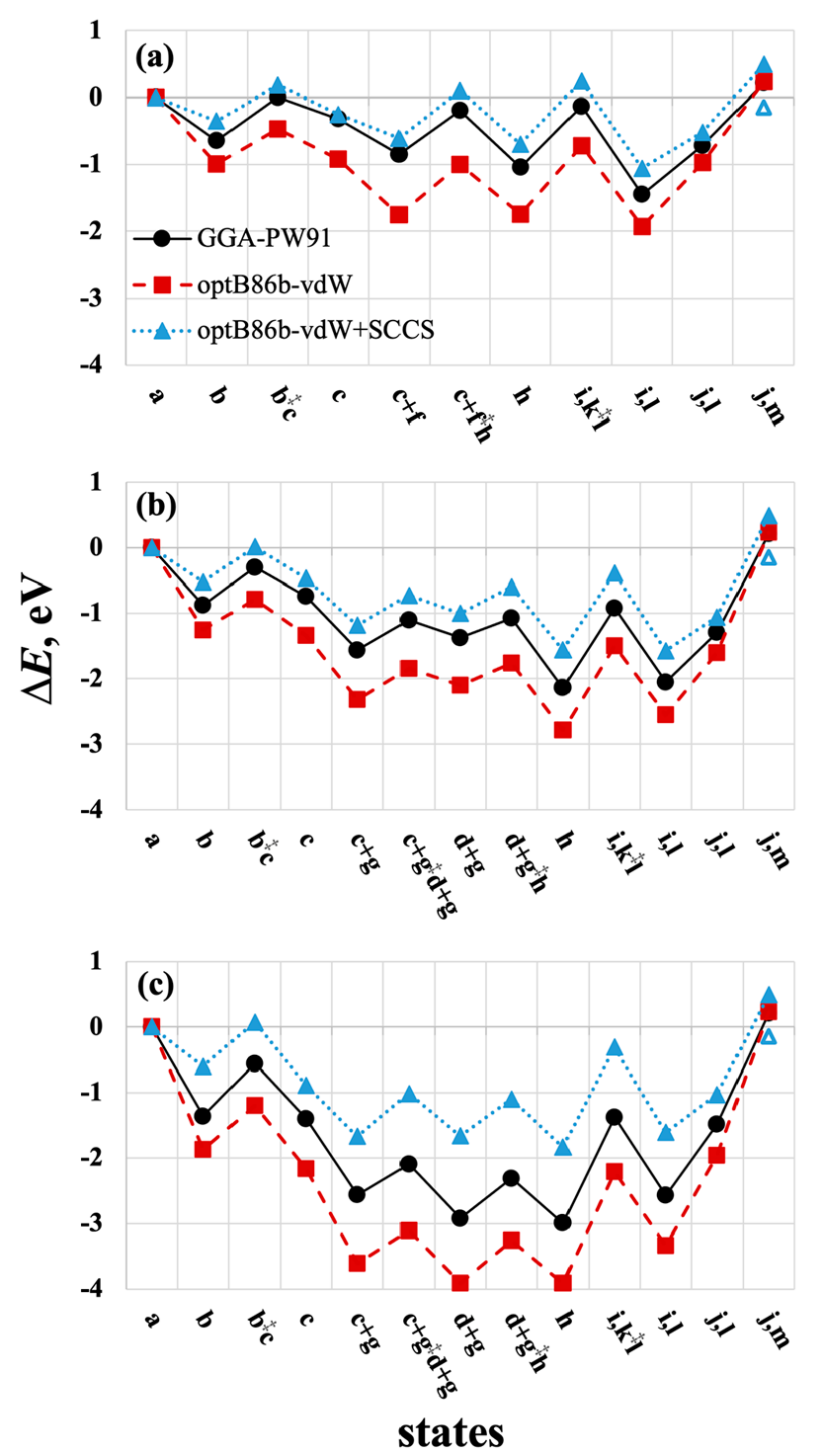
Minimum-energy reaction energy profiles for deamidation and hydrolysis of acetamide on (111), (110), and (100) facets (a–c) respectively, calculated using GGA-PW91 (black circles, VASP), optB86b-vdW (red squares, QE), and optB86b-vdW+SCCS (blue triangles, QE). The reaction states are (a) acetamide, water; (b) η^1^ acetamide, (b^‡^c) TS of nucleophilic attack by O_latt_; (c) TI; (c + f) TI + H_2_O; (c + f^‡^h) TS of C–N scission + H_2_O; (c + g) TI + OH + H; (c + g^‡^d + g) TS of C–N scission + OH + H; (d + g) acetyl + NH_2_ + OH + H; (d + g^‡^h) TS of NH_2_ hydrogenation; (h) acetyl + NH_3_ + OH; (i, k^‡^l) NH_3_, TS of OH attack; (i, l) NH_3_, acetate + H, (j, l) NH_3_ desorbed; and (j, m) acetic acid desorbed. Labels correspond to those in Table 2. Hollow blue triangle in each panel corresponds to aqueous NH_4_^+^ and CH_3_COO^–^. The optB86b-vdW profile, when calculated using VASP, differs from QE (red squares) by 0.11 eV or less.

Based on the literature, we propose and investigate the following mechanism for the deamidation and hydrolysis of acetamide, which represents a direct reaction pathway to the products, acetic acid and ammonia. The steps include (1) adsorption of acetamide in the η^1^ state and then the TI; (2) C–N scission forming acetyl and NH_2_; (3) adsorption of H_2_O; (4) hydrogenation of NH_2_ forming NH_3_; (5) attack of acetyl by OH forming acetate; (6) desorption of NH_3_ and acetic acid. The reaction parameters for each of these steps on the three low-index CeO_2_ facets calculated using GGA-PW91 are tabulated in Table 2. The snapshots of the intermediate states on the (111) facet are shown in Figure 4, and those on the (110) and (100) facets are shown in Figures S2 and S3, respectively, in the Supporting Information. The minimum-energy reaction energy profiles based on the intermediate states and elementary steps proposed in Table 2 are mapped out in Figure 5. Since free-energy contributions at the gas/solid interface are expected to be minor for reactions of small organic compounds on ceria under ambient conditions,^84^ they are omitted from Figure 5.

**Table 2:** GGA-PW91 Activation Barrier (*E*_a_) and Reaction Energy (Δ*E*_rxn_) for the Proposed Elementary Steps in Deamidation and Hydrolysis of Acetamide on the Three Low-Index CeO_2_ Facets

		(111)		(110)		(100)	
label	elementary step^a^	*E*_a_ (eV)	Δ*E*_rxn_ (eV)	*E*_a_ (eV)	Δ*E*_rxn_ (eV)	*E*_a_ (eV)	Δ*E*_rxn_ (eV)
a → b	AcD(g) → η^1^-AcD	0^b^	–0.64	0^b^	–0.88	0^b^	–1.36
b → c	η^1^-AcD → TI	0.65	+0.32	0.57	+0.14	0.80	–0.04
c → d	TI → Ac + NH_2_	0.73	+0.62	0.42	+0.19	0.52	–0.25
e → f	H_2_O(g) → H_2_O	–b	–0.53	–b	–0.78	–b	–0.95
f → g	H_2_O → OH + H	0.11	+0.02	0.38	–0.22	0.02	–0.64
c + f → h	TI + H_2_O → Ac + NH_3_ + OH	0.65	–0.20	–	–	–	–
c + g → d + g	TI + OH + H → Ac + NH_2_ + OH + H	0.84	+0.82	0.46	+0.19	0.47	–0.36
d + g → h	Ac + NH_2_ + OH + H → Ac + NH_3_ + OH	–c	–	0.30	–0.76	0.36	–0.07
i → j	NH_3_ → NH_3_(g)	0.74^b^	+0.74	0.76^b^	+0.76	1.09^b^	+1.09
k → l	Ac + OH → acetate + H	0.17	–0.42	0.23	–0.14	0.78	+0.67
l → m	acetate + H → AcA(g)	0.93^b^	+0.93	1.51^b^	+1.51	1.70^b^	+1.70

a AcD = acetamide; Ac = acetyl; AcA = acetic acid.

b Adsorption is assumed to be barrier-less and desorption is assumed to have no kinetic barrier in addition to a thermodynamic barrier.

c OH and H recombine to form water before hydrogen transfer to NH_2_.

A comparison of the calculated reaction energy profiles for the same overall reaction in gas phase, AcD_(g)_ + H_2_O_(g)_ → NH_3(g)_ + CH_3_COOH_(g)_ (black lines, Figures 5a–c), reveals a progressively deepening energy landscape from (111) to (110) and to (100). The most stable state on each facet is either h (acetyl + NH_3_ + OH) or i, l (NH_3_, acetate + H), meaning that the reaction is limited by product desorption, at least in the gas phase.

The *E*_a_ value for C–N bond scission in the TI (step c → d) producing acetyl and NH_2_ is 0.73 eV on (111), and less on (100) and (110). The TS of C–N scission is higher in energy than gas-phase acetamide (i.e., zero on Δ*E* axis) on (111), meaning that it is more likely for the molecule to desorb than to undergo decomposition on this facet, according to GGA-PW91. The step is endothermic by +0.62 eV on (111) but mildly exothermic on (100) (−0.25 eV). The C–N distance at the TS is 2.25 Å on (111) (Figure 4c^‡^d), 2.14 Å on (110) (Figure S2c^‡^d), and 2.06 Å on (100) (Figure S3c^‡^d). The resulting acetyl group binds to O_latt_ via its carbonyl C. Different from the mononuclear Zn^2+^ active center in CPA and thermolysin,^13,14^ the (110) and (111) facets (cf. Figure 2) each provide a pair of Ce^4+^ cation sites at a suitable distance and angle with respect to O_latt_ (the nucleophile attacking the carbonyl C) that can coordinate to both the O and N atoms in acetamide simultaneously in the TS. A parallel situation is found in ester decomposition on metal surfaces, where a triangular ensemble of three metal atoms activates the C(=O)–O bond.^99^

The situation is different on the (100) facet where Ce^4+^ and O_latt_ atoms are linearly aligned, so that in the TI and TS of C–N bond scission, the entire dioxo complex must shift to a nearest br site to better accommodate the rigid O–C–N angle (see Figures S3c and S3c^‡^d). A much larger barrier exists for the TI to enolize at its methyl end (1.60 eV on (111)), so that reaction pathway is not considered further.

Akin to what has been reported for coadsorbed pairs of open-shell molecules on oxides that have large band gaps,^100−102^ cooperative interaction is notable between the molecular fragments produced from C–N bond scission, acetyl, and NH_2_, on all three facets, being −0.88, −1.26, and −1.27 eV on the (111), (110), and (100) facets (see Table S2 in the Supporting Information). Therefore, the two fragments are expected to remain in close vicinity of each other at mild temperatures, and NH_2_ hydrogenation to NH_3_ is investigated in the presence of acetyl.

In agreement with previous reports,^103−105^ we find water dissociation by itself to have small barriers on all three facets (Table 2, step e → f). On (111), coadsorption with water lowers the *E*_a_ of C–N bond scission by ca. 0.1 eV, and a coadsorbed water molecule nearly spontaneously transfers a hydrogen atom to NH_2_ forming NH_3_ and OH so hydrogen transfer is included as part of step c + f → h in Table 2. A water molecule in close vicinity of acetamide dissociates spontaneously on (110) and (100), although C–N bond scission that occurs in the presence of a pair of OH and H (step c + g → d + g) on these two facets does not differ much from C–N bond scission in the absence of water (cf. step c → d). C–N bond scission is followed by NH_2_ accepting the dissociated H atom to forming NH_3_ (step d + g → h), which has an smaller *E*_a_ still than the preceding step. The energetic difference between the NH_2_ and NH_3_ states is the smallest on the (100) facet (Figure 5), making it more likely for the coverage of NH_2_ to build up and become detectable on (100).

The acetyl and OH groups stabilize each other strongly, like acetyl and NH_2_ (Table S2). The OH group attacks acetyl to form an η^1^ acetate coordinated to an H atom (step k → l), i.e., CH_3_COO–HO_latt_, which amounts to a partially dissociated acetic acid. This state is iso-energetic to the μ acetate coadsorbed with an H atom on an adjacent O_latt_ reported in ref (71). This hydroxylation step has a small *E*_a_ (0.17 and 0.23 eV) and exothermic Δ*E*_rxn_ on (111) and (110), but is endothermic with a sizable *E*_a_ (0.78 eV) on (100). The minimum-energy path for this step on all three facets involves the OH group displacing the O_latt_ atom to which acetyl is bonded, that is, in a sense, the OH group pushes an acetate group (i.e., acetyl–O_latt_) out of the lattice site that it occupies (cf. Figure 4k) and heals the oxygen vacancy that is left behind. Both NH_3_ and acetic acid adsorb on the ceria surfaces strongly (cf. Table S1) so that the desorption of the product molecules to the gas phase, particularly acetic acid, (l → m, Table 2) is more rate-limiting than all of the surface elementary steps. This is more so on the (110) and (100) facets than on (111).

We further observe that the reverse of steps l → m, k → l, f → g, and e → f constitutes a reaction channel for acetic acid to reduce the ceria surfaces, which is energetically competitive with acetic acid desorption (Table S3 in the Supporting Information). By removing OH and H as water, a mixture of acetyl and acetate (the other species in step k → l), and not solely acetate, would populate the surface. This surface dehydration channel is not expected to be favorable in aqueous phase based on Le Chatelier’s principle, but may become operative when the availability of water is low or nill. This may explain the evolution of water from CeO_2_(111) at near-ambient temperature following acetic acid adsorption in the TPD experiments of Calaza et al.^71^

Although the main reaction considered here is the hydrolysis of acetamide, it is worthwhile to remark that an alternate, dehydration pathway exists for acetamide also; see discussion in the Supporting Information. The dehydration of acetamide is energetically not competitive with the deamidation and hydrolysis of acetamide but likewise could become favorable under dry conditions at elevated temperatures.

### 3.3 Effects of vdW Interaction and Solvation

Our prior work^52,84^ suggests that vdW interactions contribute to the stability of organic molecules as small as acetaldehyde when they are adsorbed on ceria. Thus, we have recalculated the minimum-energy reaction energy profiles on the three low-index facets using the optB86b-vdW functional (red dashed traces, Figures 5a–c). Each profile is deepened by vdW interactions on the Δ*E* axis, particularly for the states in which two molecules are coadsorbed.

Biochemically, deamidation reactions occur in aqueous environments. For ceria functioning as mimics of natural enzymes, an additional possibility of ionized products (NH_4_^+^ and CH_3_COO^–^) exists since ammonia and acetic acid are mildly basic and acidic, respectively. We have calculated the free energy of hydration (Δ*G*_hyd_) for the reactant and product species, using VASP/VASPsol and QE/Environ. The results are tabulated in Table S4 in the Supporting Information. It can be seen that the two implicit solvation models agree closely with each other, irrespective of the exchange-correlation functional used. For neutral, closed-shell species, our results also agree closely with the values calculated based on the Langevin dipoles solvation model by Warshel and co-workers and with experimental measurements.^106,107^ Noticeable deviations are seen for the ionic species, with the theoretical models, particularly VASPsol and Environ, underpredicting solvation for the acetate anion and overpredicting it for the ammonium cation. Since ionic species carry localized charges that are strongly solvated by water, a lack of explicitly description of localized hydrogen bonding by the implicit solvation models is expected to lead to some errors. While the overall deamidation reaction is slightly endothermic and endergonic in the gas phase, the reaction AcD(aq) + H_2_O(aq) → NH_4_^+^(aq) + CH_3_COO^–^(aq) is computed to be mildly exergonic at −0.18 eV, which is 0.31 eV lower than the Δ*G*_rxn_ for the gas phase reaction (see Table S5 in the Supporting Information). It suggests that aqueous phase is a thermodynamically necessary condition for the deamidation reaction to occur under ambient conditions. The solvated ionic final product state is indicated by a hollow triangle in Figure 5.

We have then attempted to estimate the effect of hydration on the stability of the surface intermediates in combination with vdW interactions, using the SCCS implicit solvation model implemented in QE/Environ. Solvation by water is predicted to reduce the surface energy of the clean ceria facets by 11, 21, and 25 meV/Å^2^ for the (111), (110), and (100) facets, respectively, which is large, compared to, e.g. Pt(111), for which the surface energy is reduced by implicit solvation by water by 2 meV/Å^2^.^87^ The sizable stabilization is consistent with the fact that ceria surfaces exhibit arrays of localized charge centers, which elicit a strong dielectric response by the solvent, independent of specific chemical or hydrogen bonding interactions between the aqueous phase and the surface.

The adsorption of the reactant and product molecules generally interferes with the interfacial dielectric response of aqueous phase to the ceria surfaces. The solvation of the surfaces is reduced (i.e., positive ΔΔ*G*_hyd_, Table 3) particularly for (100). The exceptions include H_2_O on (111), which has no effect on surface solvation, and acetic acid on (111) and (110), which enhances surface solvation. Viewed from a different angle, the adsorption strengths of all the molecules are reduced (i.e., positive ΔΔ*E*_ads_, Table 3), meaning more facile desorption in the aqueous phase than in the gas phase.

**Table 3:** Various Measures of Solvation Effects on Reactant and Product Molecules, Calculated Using optB86b-vdW with QE^a^

		(111)				(110)				(100)			
	Δ*G*_hyd_ (aq)	Δ*E*_ads_	Δ*E*_ads_^SCCS^	ΔΔ*G*_hyd_	ΔΔ*E*_ads_	Δ*E*_ads_	Δ*E*_ads_^SCCS^	ΔΔ*G*_hyd_	ΔΔ*E*_ads_	Δ*E*_ads_	Δ*E*_ads_^SCCS^	ΔΔ*G*_hyd_	ΔΔ*E*_ads_
η^1^-AcD	–0.52	–0.99	–0.35	+0.12	+0.64	–1.25	–0.52	+0.21	+0.73	–1.87	–0.60	+0.75	+1.27
H_2_O	–0.36	–0.69	–0.32	+0.01	+0.37	–0.99	–0.46	+0.17	+0.53	–1.19	–0.48	+0.35	+0.71
NH_3_	–0.21	–0.96	–0.54	+0.21	+0.43	–0.95	–0.51	+0.23	+0.44	–1.37	–0.58	+0.58	+0.80
acetate + H	–0.41	–1.20	–1.01	–0.22	+0.19	–1.83	–1.55	–0.12	+0.28	–2.19	–1.52	+0.27	+0.67

a Acetic acid is present as an acetate + H pair on the three facets. Differences in optB86b-vdW Δ*E*_ads_ calculated using VASP (reported in Table S1) vs QE are 0.11 eV or less in all cases. Δ*G*_hyd (aq)_ = free energy of hydration for molecules in aqueous phase; ΔΔ*G*_hyd_ = change in free energy of hydration for surface with adsorbate vs bare surface; ΔΔ*E*_ads_ = decrease in adsorption strength due to hydration, i.e., amount by which desorption becomes more facile; ΔΔ*E*_ads_ = ΔΔ*G*_hyd_ – Δ*G*_hyd (aq)_ = Δ*E*_ads_^SCCS^ – Δ*E*_ads_

Overall, the proposed pathway has a shallower reaction energy profile on (111) than on (110) and (100) when both vdW interaction and solvation effects are taken into account, suggesting the (111) facet to be the most advantageous of the three facets for catalyzing acetamide hydrolysis in aqueous phase based on thermodynamics. TS calculations are not repeated with vdW interaction and solvation effects included due to convergence difficulties. Instead, we make the assumption that the vdW and solvation effects on a TS are the average of these effects on the IS and FS of an elementary step. This approach would yield errors of 0–0.15 eV for the stability of TS based on the results of our previous study on aldol condensation of acetaldehyde on CeO_2_(111).^84^ Under this assumption, conversion from the η^1^ state to the TI is seen to be slightly outcompeted by acetamide desorption on all three facets, of which the (110) facet is the most efficient at directing a given molecular flux toward acetamide deamidation versus acetamide desorption, followed by (100), and then (111). On the other hand, the adsorption energy of acetic acid (Δ*E*_ads_^SCCS^, Table 3) remains at −1 eV on (111) and lower on (110) and (100) (Δ*E*_ads_^SCCS^, Table 3), suggesting that product desorption continues to be limiting, particularly on the two more open facets, even when assisted by an aqueous phase. A strategy that modifies the C–N bond scission activity and acetate adsorption strength differently will be needed to further optimize an oxide-based catalyst including ceria for the reaction. Once desorbed, acetic acid and NH_3_ can undergo acid–base neutralization by ionizing, which provides the thermodynamic driving force for the overall reaction.

### 3.4 Decomposition of Amides, Amidines, and Generalized Esters on Ceria

**Figure 6:**
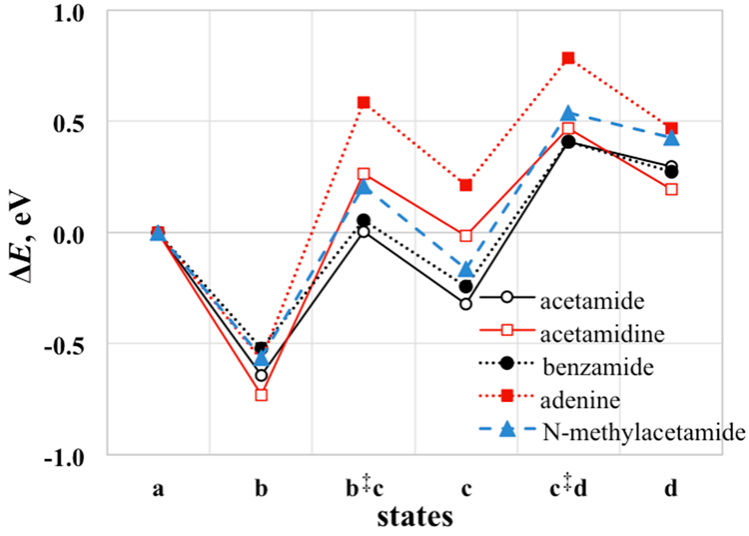
GGA-PW91 reaction energy profiles up to C–N bond scission in various amine compounds on CeO_2_(111). The surface reaction states are (a) gas-phase molecule, (b) η^1^ state, (b^‡^c) TS of nucleophilic attack by O_latt_, (c) TI, (c^‡^d) TS of C–N scission, and (d) C−N scission products.

The activation and dissociation of the C–N bond over Ce–O_latt_–Ce sites are not limited to acetamide. We demonstrate this for several other amine compounds, including benzamide, *N*-methylacetamide, acetamidine, and adenine. The reaction energy profiles up to C–N bond scission for these amine compounds on CeO_2_(111) are plotted in Figure 6 for comparison with acetamide. The immediate fragments that result are benzoyl + NH_2_ (for benzamide), acetyl + CH_3_NH (for *N*-methylacetamide), CH_3_CNH + NH_2_ (for acetamidine), and purine + NH_2_ (for adenine). These fragments also mutually stabilize on the surface (Table S2). Secondary or tertiary amides (e.g., *N*-methylacetamide) produce amines instead of ammonia.

Benzamide and *N*-methylacetamide can be viewed as derivatives of acetamide. Benzamide has the methyl group in acetamide replaced with a phenyl group, whereas *N*-methylacetamide is obtained by replacing one of the amine hydrogen atoms with a methyl group, making it perhaps the simplest model for peptide bonds in proteins.^108^ Acetamidine is a simple amidine, an imine analog of an amide with the C=O group replaced with a C=NH group. These molecules are likewise open to nucleophilic attack by O_latt_ of ceria at the carbonyl C to form TI and then undergo C–N bond scission, with *E*_a_ similar to that for acetamide (Figure 6) except for one.

Previously, we studied the adsorption of a primary nucleobase, adenine, on CeO_2_(111) as a model system for organic/inorganic interfaces.^52^ Adenine can be viewed as a cyclic amidine in which the C=NH group is a part of an aromatic ring. In nature, adenine deaminase catalyzes the conversion of adenine to NH_3_ and hypoxanthine. The enzyme contains a binuclear metal center of two Fe^2+^, which stabilizes a hydroxide group that attacks the C6 position of adenine, the carbon atom to which −NH_2_ is attached, and replaces the amine group.^92^ On CeO_2_(111), because of the aromaticity of the purine group, nucleophilic attack by O_latt_ at the C6 position forming TI has a notably higher *E*_a_ value (1.16 eV) than in the other compounds, but is followed by a more modest *E*_a_ value for C–N bond scission than in acetamide (0.48 eV).

**Figure 7:**
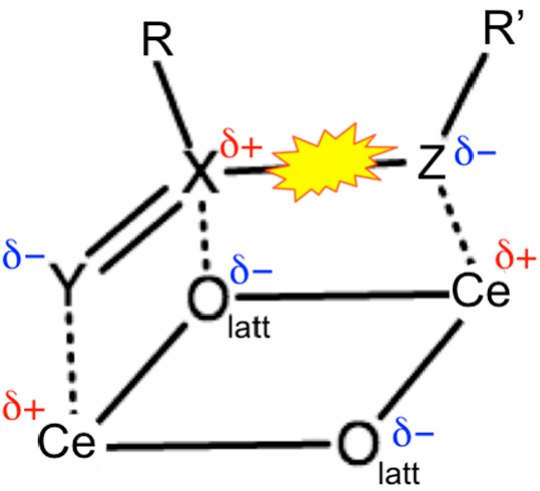
Schematic illustrating how a generalized ester (RX(=Y)–ZR′) interacts with an ensemble of Ce–O_latt_–Ce surface sites. The generalized ester bond is highlighted.

**Figure 8:**
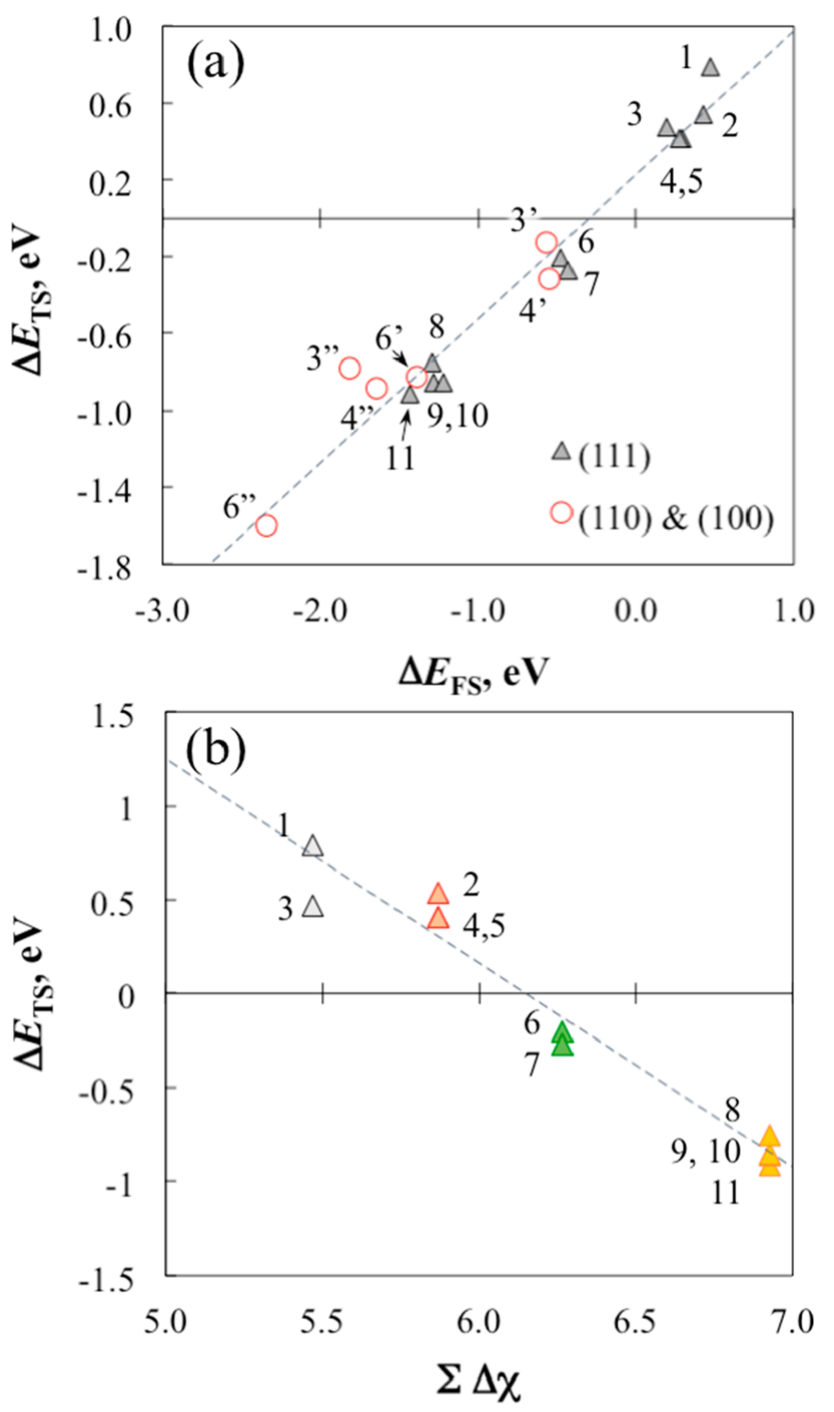
(a) GGA-PW91 stability of the TS versus the FS (coadsorbed dissociated fragments) of the scission of X–Z generalized ester bond, and (b) stability of the TS versus the electronegativity-based descriptor, ∑Δχ, for several GEs on CeO_2_(111). The compounds included are adenine (1), *N*-methylacetamide (2), acetamidine (3), acetamide (4), benzamide (5), methyl acetate (6), methyl formate (7), chloromethyl phosphate (8), *para*-chlorophenyl phosphate (9), and phenyl phosphate (10), and *para*-nitrophenyl phosphate (11). Unmarked numbers indicate results on (111), single prime symbol (′) indicates results on (110), and double prime symbol (′′) indicates results on (100). The TS and FS of the phosphates are those that do not involve spontaneous dissociation of an acidic proton. Data values are listed in Table S6 in the Supporting Information, and snapshots of the TS are shown in Figure S4 in the Supporting Information. The lines are results of linear regression: in panel (a), Δ*E*_TS_ = 0.750 Δ*E*_FS_ + 0.226, *R*^2^ = 0.958; in panel (b), Δ*E*_TS_ = −1.086 ∑Δχ + 6.677, *R*^2^ = 0.956 with *a* = 1.83.

The C–N bond scission steps for acetamide, benzamide, *N*-methylacetamide, acetamidine, and adenine are analyzed in terms of a linear energy relationship between the TS and the FS.^109,110^ To this dataset, we also add the results for several other examples of generalized esters (GEs), which all contain the X(=Y)–Z moiety, where Y and Z are more electronegative atoms than X, resulting in a polarized X–Z generalized ester bond (Figure 7). The additional GEs include methyl formate and methyl acetate (X=C, Y=Z=O), and phenyl phosphate, *para*-nitrophenyl phosphate, *para*-chlorophenyl phosphate, and chloromethyl phosphate (X=P, Y=Z=O) that are taken from our previous work on phosphate monoesters on CeO_2_(111).^94,95^ The results for acetamide, acetamidine, and methyl acetate on the (110) and (100) facets are also included, for which the dissociated fragments of RX=Y and ZR′ are notably more stable than on (111). When the stability of the TS of the X–Z bond scission is plotted against that of the FS for this diverse group of GEs, a linear Brønsted–Evans–Polanyi relationship is obtained (Figure 8a). The TS for the carboxylates and phosphates on (111), and for all the compounds considered on (110) and (100), lie below 0 eV versus the gas phase, which suggests ceria to be a potentially effective catalyst for de-esterification of such compounds. This is consistent with previous theoretical studies that report low *E*_a_ for the scission of ester bonds in other examples of GEs including dimethylcarbonate^93^ and dimethyl methylphosphonate.^111^ Using this linear relationship, one may quickly estimate the stability of the TS of the scission of the generalized ester bond in GEs.

The data in Figure 8a cluster in separate groups for phosphates, carboxylates, and amides/amidines on a given facet. This clustering hints at underlying, systematic factors that influence the stability of the surface complexes that these organic compounds form on ceria. For acetamide and the related compounds, the carbonyl C–O_latt_ bond obviously plays an important role in the stability of the TS of C–N bond scission on the ceria surfaces. At the same time, the carbonyl O and the amine N atoms are coordinated to adjacent Ce sites, but how strong the O(N)–Ce interaction is versus the C–O_latt_ bond is unclear. We surmise that the nature of the X, Y, and Z atoms in a GE that correspond to the ensemble of Ce–O_latt_–Ce surface sites determines the stability of the TS for a given GE on CeO_2_(111). Inspired by Capdevila-Cortada et al.,^96^ we propose a descriptor to measure this interaction, which is based on the electronegativity (χ_*i*_) of the atoms involved:
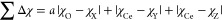
We use the Pauling electronegativity for the elements involved, i.e., χ_Ce_ = 1.12, χ_C_ = 2.55, χ_N_ = 3.04, χ_O_ = 3.44, and χ_P_ = 2.19. *a* is an adjustable parameter to represent different weighting of the X–O_latt_ bonding relative to the interaction between the Y and Z atoms and Ce. The stability of the TS for the scission of the X–Z generalized ester bond on CeO_2_(111) included in Figure 8a is replotted against ∑Δχ in Figure 8b. While this simple descriptor does not account for the chemical environment beyond the X(=Y)–Z moiety or the catalytic site structure, Figure 8b suggests that it captures the trend in the stability of the TS, which supports our hypothesis. The optimized value of *a* is 1.83, indicating that the X–O_latt_ bond is notably stronger than the interaction between the Y or Z atom and Ce^4+^. The different coordination environment and geometry of the Ce–O_latt_–Ce ensemble would account for the different reactivity of the facets toward the decomposition of GEs.

Of the two fragments that are produced upon scission of the generalized ester bond, the ZR′ fragment is hydrogenated into an alcohol or amine/NH_3_, while the RX(=Y) fragment is hydroxylated into RX(=Y)OH that is tautomeric with RX(=O)YH. Conceivably, if RX(=Y)OH is not tied up in an aromatic moiety (e.g., adenine), it could undergo hydrolysis again until all X–Y bonds (when Y ≠ O) are replaced by X–O bonds to produce the corresponding acid (RXOOH); e.g., acetamidine fully hydrolyzed to acetic acid. Based on the examples used in Figure 8, we further compare the Δ*E*_ads_ values of several carboxylic acids, including formic, acetic, and benzoic acids, on CeO_2_(111). The difference in Δ*E*_ads_ values turns out to be noticeable in the gas phase but less pronounced at the water/solid interface (see Figure S5 in the Supporting Information).

## 4 Conclusions

Ceria has been regarded as enzyme-mimicking because it exhibits catalytic activity under ambient conditions for the decomposition via hydrolysis of a range of organic compounds including carboxylates, phosphates, and amides, which is reminiscent of the action of natural metallohydrolases, including phosphatase and peptidase, that use metal ions to catalyze the hydrolysis of many biological compounds of the same types. We have performed periodic DFT calculations to investigate the mechanism and energetics of deamidation via hydrolysis on the three low-index CeO_2_ facets, (111), (110), and (100), using acetamide as the main model compound.

The reaction shares similar features on the three facets, of which (111) is the least reactive and binds reaction intermediates least strongly while (100) is the most reactive. Acetamide adsorbs molecularly with its carbonyl O coordinated to a Ce cation and one of its amine H coordinated to an adjacent lattice O anion. The amide C–N bond scission is preceded by the formation of a tetrahedral intermediate (TI) state, formed via nucleophilic attack by lattice O on the carbonyl C, which has an activation barrier of *E*_a_ = 0.65, 0.57, and 0.80 eV on the (111), (110), and (100) facets, respectively. In the presence of a water molecule, C–N scission in the TI occurs with a more modest barrier (*E*_a_ = 0.65, 0.46, and 0.47 eV on (111), (110), and (100)), producing acetyl and NH_2_ fragments. NH_2_ readily extracts a hydrogen atom from water to form NH_3_. The remaining OH group attacks acetyl to form an acetate + H pair, which is facile on (111) (*E*_a_ = 0.17 eV) but has a more substantial barrier on (100) (*E*_a_ = 0.78 eV). The overall reaction is desorption-limited in the gas phase, particularly for acetic acid on the two more open facets. The calculated desorption energies for NH_3_ and acetic acid are 0.74 and 0.93 eV on (111), 0.76 and 1.51 eV on (110), and 1.09 and 1.70 eV on (100). These values are larger than the calculated *E*_a_ values for all the surface reaction steps in the minimum-energy reaction mechanism on the respective facets.

Since biologically and environmentally relevant deamidation reactions occur in the aqueous phase, we have further used the SCCS implicit solvent model in combination with van der Waals corrections to estimate the reaction energetics at the aqueous interface. While vdW interactions stabilize the surface intermediates, the implicit solvent has the opposite effect and makes the reaction energy profiles less corrugated than in the gas phase. Desorption of all reactant and product species is predicted to be enhanced by the aqueous phase, although the desorption of acetic acid is expected to remain kinetically hindered at ambient temperature.

A survey of the literature and our own studies suggest that the Ce–O_latt_–Ce site ensembles present on ceria surfaces are well-suited to activating organic moieties of the X(=Y)–Z type, in which Y and Z are more electronegative atoms than X, resulting in a polarized X–Z bond. We term such compounds generalized esters (GEs), which includes carboxylates, phosphates, and amides among many others. A simple descriptor (∑Δχ) is proposed that takes into account the electronegativity of both the atoms constituting the bond (X, Z) and those of the catalytic site (Ce, O). It captures a linear trend in the stability of the TS of X–Z bond scission on CeO_2_(111), revealing a connection between the composition of the oxide surface and the catalytic activity for the decomposition of GEs.

Our work identifies certain factors that will need to be addressed in future catalyst design based on ceria and other metal compounds for catalytic decomposition of a wide range of organic and biologically active compounds, such as those controlling the catalytic activity for cleaving generalized ester bonds and the rate-limiting desorption of carboxylate products. The findings are also relevant to ceria- and oxide-catalyzed generalized esterification and trans-esterification reactions, which involve the formation instead of scission of generalized ester bonds.^46,93,112^

## Supporting Information Available

Comparison of adsorption energies of reaction reactants and products on CeO_2_(111), (110), and (100) with literature values; interaction energies of reaction intermediates on CeO_2_(111), (110), and (100); comparison of reaction energies for the desorption of water compared to desorption of acetic acid on CeO_2_(111), (110), and (100); comparison of free energies of hydration of reactant and product species with literature values; overall total and free energies for acetamide hydrolysis; tabulated data used in Figure 8; simulated vibrational spectra of acetamide adsorbed on CeO_2_(111), (110), and (100); snapshots of stable intermediates and transition states of the elementary steps in deamidation and hydrolysis of acetamide on stoichiometric CeO_2_(110) and (100) facets; snapshots of transition states of X–Z bond scission in GEs on CeO_2_(111), (110), and (100); adsorption energies of several carboxylic acids on CeO_2_(111); results and discussion on acetamide dehydration on CeO_2_(111), (110), and (100) (PDF)
