# Correction to “Hydrolysis of Acetamide on Low-Index CeO_2_ Surfaces: Ceria as a Deamidation and General De-esterification Catalyst”

**DOI:** 10.1021/acscatal.3c02062

**Published:** 2023-05-23

**Authors:** Suman Bhasker-Ranganath, Ye Xu

Figure 5 of the original article contains an error. The error was due to the use of gas-phase NH_3_ in the calculation of state (i,k^‡^l). Adsorbed NH_3_ should have been used instead because this state occurs prior to NH_3_ desorption. The error resulted in much larger activation barriers for OH attack on acetyl being depicted in the original Figure 5 for the three facets of ceria. They appear comparable to the barriers of acetic acid desorption, which is inconsistent with the conclusion that product desorption is rate-limiting in this reaction at ambient temperature.

**Figure 5:**
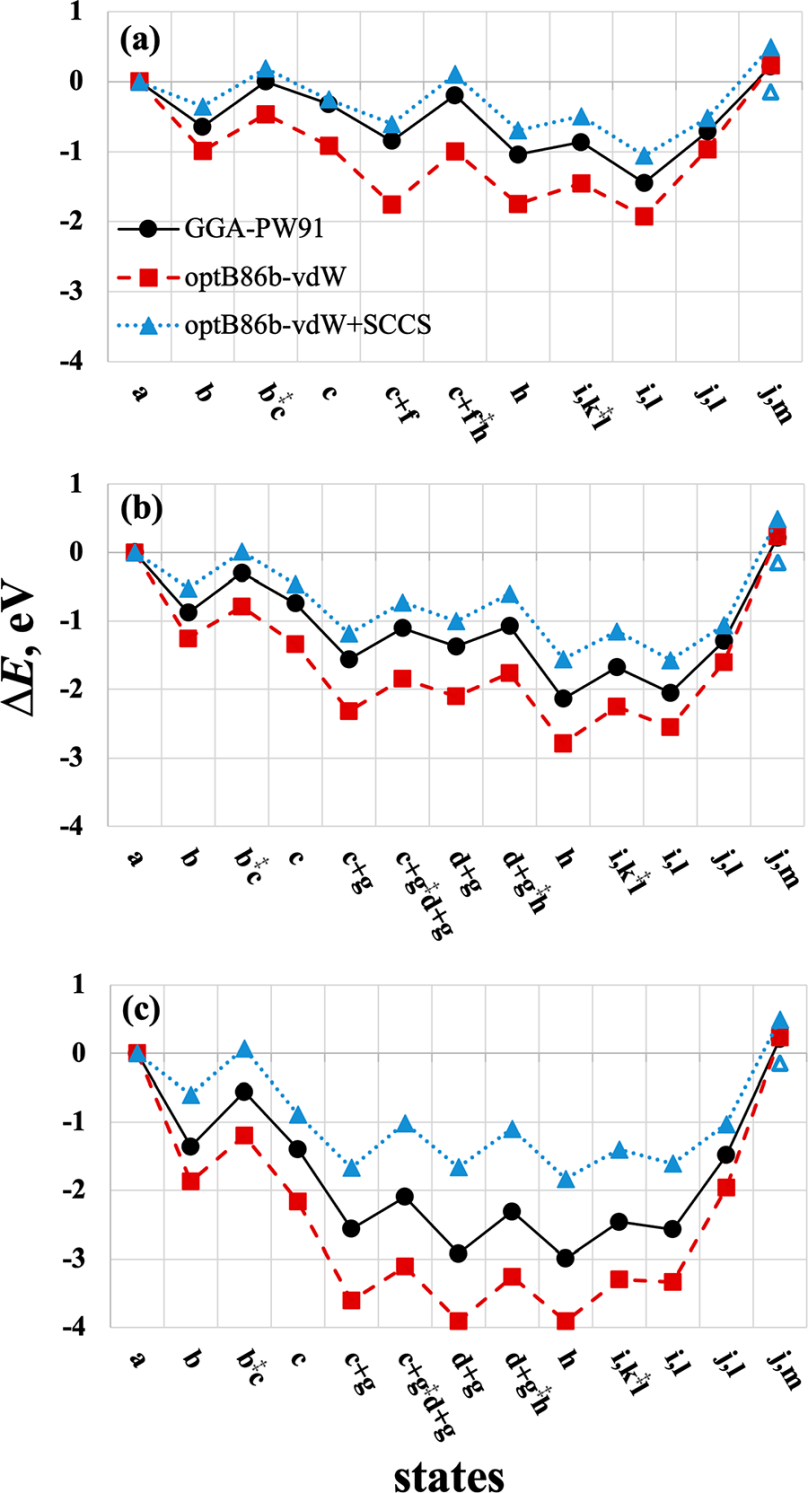
Minimum-energy reaction energy profiles for deamidation and hydrolysis of acetamide on (111), (110), and (100) facets (a-c) respectively, calculated using GGA-PW91 (black circles, VASP), optB86b-vdW (red squares, QE), and optB86b-vdW+SCCS (blue triangles, QE). The reaction states are (a) acetamide, water; (b) η^1^ acetamide; (b^‡^c) TS of nucleophilic attack by O_latt_; (c) TI; (c+f) TI + H_2_O; (c+f^‡^h) TS of C–N scission + H_2_O; (c+g) TI + OH + H; (c+g^‡^d+g) TS of C–N scission + OH + H; (d+g) acetyl + NH_2_ + OH + H; (d+g^‡^h) TS of NH_2_ hydrogenation; (h) acetyl + NH_3_ + OH; (i,k^‡^l) NH_3_, TS of OH attack; (i,l) NH_3_, acetate + H; (j,l) NH_3_ desorbed; and (j,m) acetic acid desorbed. Labels correspond to those in Table 2. Hollow blue triangle in each panel corresponds to aqueous NH_4_^+^ and CH_3_COO^–^. The optB86b-vdW profile, when calculated using VASP (not shown), differs from QE (red squares) by 0.11 eV or less.

The corrected version is shown below with the same caption as before. State (i,k^‡^l) is now revised to include NH_3_ adsorbed at infinite separation from the transition state k^‡^l. As can be seen, and as can be verified with entry k → l, Table 2 of the original article, the OH attack is not a kinetically relevant step in this reaction. All other states in Figure 5 have the correct energies.

