# Supplementary material for: Hydrolysis of Acetamide on Low-Index CeO2 Surfaces: Ceria as a Deamidation and General De-esterification Catalyst
Source: ACS Catal. 2022 Aug 5;12(16):10222–34. doi: 10.1021/acscatal.2c02514 (PMC9397537; doi:10.1021/acscatal.2c02514)
Supplement: Supplementary file 1 — cs2c02514_si_001.pdf [file cs2c02514_si_001.pdf]

Hydrolysis of acetamide on low-index CeO_2 surfaces: Ceria as a deamidation and general de-esterification catalyst

*Suman Bhasker-Ranganath and Ye Xu**

Cain Department of Chemical Engineering, Louisiana State University,

Baton Rouge, LA 70803, USA

Supporting Information

* Corresponding Author. Email address: yexu@lsu.edu (Y. Xu).

Table S1. Adsorption energies (ΔE_{ads}, in eV) of reactant and product molecules on the three low-index CeO_2 facets, compared with available values calculated using comparable methods in the literature.

	(111)		(110)		(100)	
	this work	Literature	this work	literature	this work	literature
η[1]-AcD	–0.64 [†]	-	–0.88 [†]	-	–1.36 [†]	-
	–1.00 [#]		–1.31 [#]		–1.99 [#]	
TI-AcD	–0.32 [†]	-	–0.74 [†]	-	–1.40 [†]	-
	–0.95 [#]		–1.41 [#]		–2.27 [#]	
NH_3	–0.74 [†]	–0.70 [†1]	–0.76 [†]	–0.73 [†1]	–1.09 [†]	–0.61 [†1]
	–0.96 [#]	–0.66 [§2]	–1.00 [#]	–1.41 [§2]	–1.42 [#]	–1.00 [§2]
		–0.53 [§3]				
		–0.19 [†4]				
acetate + H	–0.94 [†]	–0.95 [†5]	–1.51 [†]	-	–1.71 [†]	-
	–1.21 [#]		–1.85 [#]		–2.22 [#]	
H_2O	–0.53 [†]	–0.63 [§6]	–0.78 [†]	–0.79 [§6]	–0.95 [†]	–1.28 [§6]
	–0.68 [#]	–0.58 [§7]	–1.06 [#]	–0.85 [§7]	–1.25 [#]	–1.00 [§7]
		–0.54 [§8]		–0.64 [§8]		
		–0.55 [§9]				
		–0.67 [¶9]				
		–0.73 [#9]				

AcD = acetamide. [†] GGA-PW91. [§] GGA-PBE. [#] optB86b-vdW. [¶] optPBE-vdW. Results of this work presented here are calculated using VASP. Literature values were reported at various coverages.

Table S2. GGA-PW91 interaction energies (ΔE_{int}, in eV) of several combinations of proposed reaction intermediates in acetamide hydrolysis on the three low-index CeO_2 facets, and of C-N scission intermediates in other amines on CeO_2(111).

	ΔE_{int}		
Species	(111)	(110)	(100)
acetyl + NH_2	–0.88	–1.26	–1.27
(acetyl + OH) + NH_3	–0.07	–0.23	+0.09
(acetate + H) + NH_3	–0.06	–0.21	+0.08
acetyl + OH	–1.56	–1.58	–1.50
acetate + H	–1.69	–0.99	–0.75
benzoyl + NH_2	–0.82		
acetyl + CH_3NH	–0.53		
CH_3CNH + NH_2	–0.86		
purine + NH_2	–0.87		

ΔE_{int} is the difference in DFT total energy between the species co-adsorbed vs. at infinite separation; a negative ΔE_{int} indicates the co-adsorbed state to be more stable, and vice versa.

Table S3. Comparison of GGA-PW91 reaction energies (ΔE, in eV) for water vs. acetic acid formation and desorption from the minimum-energy state in the proposed mechanism for deamidation and hydrolysis of acetamide on the three low-index CeO_2 facets.

Facet	Minimum energy state (excl. NH_3)	ΔE to water desorption	ΔE to acetic acid desorption
(111)	l (acetate + H)	$-\Delta E_{rxn}^{k\to l} - \Delta E_{rxn}^{f\to g} - \Delta E_{rxn}^{e\to f} = +0.93$	$\Delta E_{rxn}^{l\to m} = +0.93$
(110)	l (acetate + H)	$-\Delta E_{rxn}^{k\to l} - \Delta E_{rxn}^{f\to g} - \Delta E_{rxn}^{e\to f} = +1.14$	$\Delta E_{rxn}^{l\to m} = +1.51$
(100)	k (Ac + OH)	$+\Delta E_{rxn}^{k\to l} - \Delta E_{rxn}^{f\to g} - \Delta E_{rxn}^{e\to f} = +2.26$	$+\Delta E_{rxn}^{k\to l} + \Delta E_{rxn}^{l\to m} = +2.37$

Table S4. Free energies of hydration (ΔG_{hyd}, in eV) for the reactant and product species in aqueous phase calculated using VASP/VASPsol and QE/Environ.

Species	GGA-PW91 VASP	optB86b-vdW VASP	optB86b-vdW QE	literature calc.	literature exp.
AcD	–0.43	–0.43	–0.52	–0.42	–0.42
H_2O	–0.32	–0.32	–0.36	–0.39	–0.28
NH_3	–0.17	–0.17	–0.21	–0.20	–0.19
CH_3COOH	–0.33	–0.33	–0.41	–0.27	–0.29
CH_3COO^-	–2.85	–2.84	–2.82	–3.38	–3.56
NH_4^+	–3.93	–3.92	–3.95	–3.60	–3.51

ΔG_{hyd} values reported in the literature[10] are included for comparison.

Table S5. Reaction total and free energy (ΔE_{rxn} and ΔG_{rxn}, in eV) for the overall acetamide hydrolysis reaction.

	GGA-PW91 VASP	optB86b-vdW VASP	optB86b-vdW QE		
Overall reaction	$\Delta E_{rxn\ (g)}$			$\Delta G_{rxn\ (g)}$	$\Delta G_{rxn\ (aq)}$
AcD + H_2O → NH_3 + CH_3COOH	+0.22	+0.23	+0.23	+0.13	+0.47
AcD + H_2O → NH_4^+ + CH_3COO^-	+6.10	+6.08	+6.02	+5.99	–0.18

(g) and (aq) indicate gas phase and aqueous phase, respectively. ΔG_{rxn} for the gas-phase reaction is based on the optB86b-vdW results calculated using VASP and includes free energy contributions based on the vibrational energies of the molecules in the ideal gas limit at 298.15 K. ΔG_{rxn} for the aqueous-phase reaction is based on $\Delta G_{rxn\ (g)}$ and further includes the experimental free energies of hydration listed in Table S4. The standard free energy of transfer between gas and aqueous phase is assumed to be identical for each species and therefore cancels out in $\Delta G_{rxn\ (aq)}$.

Table S6. GGA-PW91 stability of the transition and final states (ΔE_{TS} and ΔE_{FS}, in eV) for dissociation of respective GEs on low-index CeO_2 facets, and the corresponding values of the electronegativity-based descriptor ($\Sigma\Delta\chi$, CeO_2(111) only).

	ΔE_{TS}	ΔE_{FS}	$\sum\Delta\chi$
(111)			
adenine	0.79	0.47	5.47
acetamidine	0.47	0.19	5.47
N-methylacetamide	0.54	0.43	5.87
acetamide	0.41	0.30	5.87
benzamide	0.41	0.27	5.87
methyl acetate	-0.21	-0.48	6.27
methyl formate	-0.27	-0.44	6.27
chloromethyl phosphate	-0.75	-1.29	6.93
phenyl phosphate	-0.86	-1.23	6.93
para-chlorophenyl phosphate	-0.86	-1.29	6.93
para-nitrophenyl phosphate	-0.91	-1.44	6.93
(110)			
acetamidine	-0.12	-0.57	
acetamide	-0.32	-0.55	
methyl acetate	-0.83	-1.40	
(100)			
acetamidine	-0.78	-1.82	
acetamide	-0.88	-1.65	
methyl acetate	-1.60	-2.34	

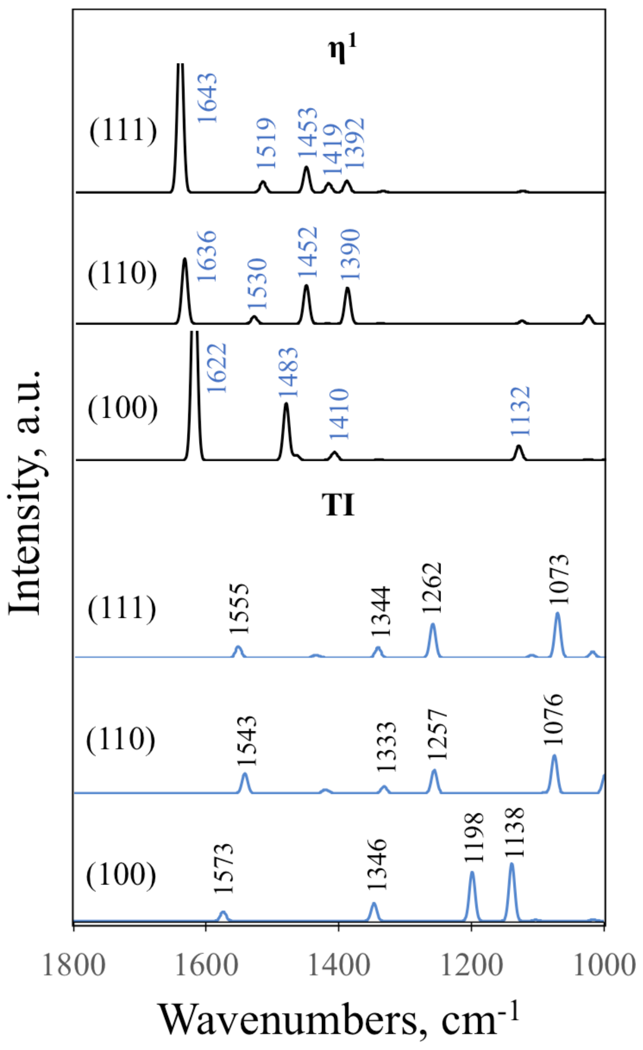

Figure S1. GGA-PW91 simulated IR spectra of acetamide in the η^1 and TI adsorbed states on the three low-index CeO_2 facets.

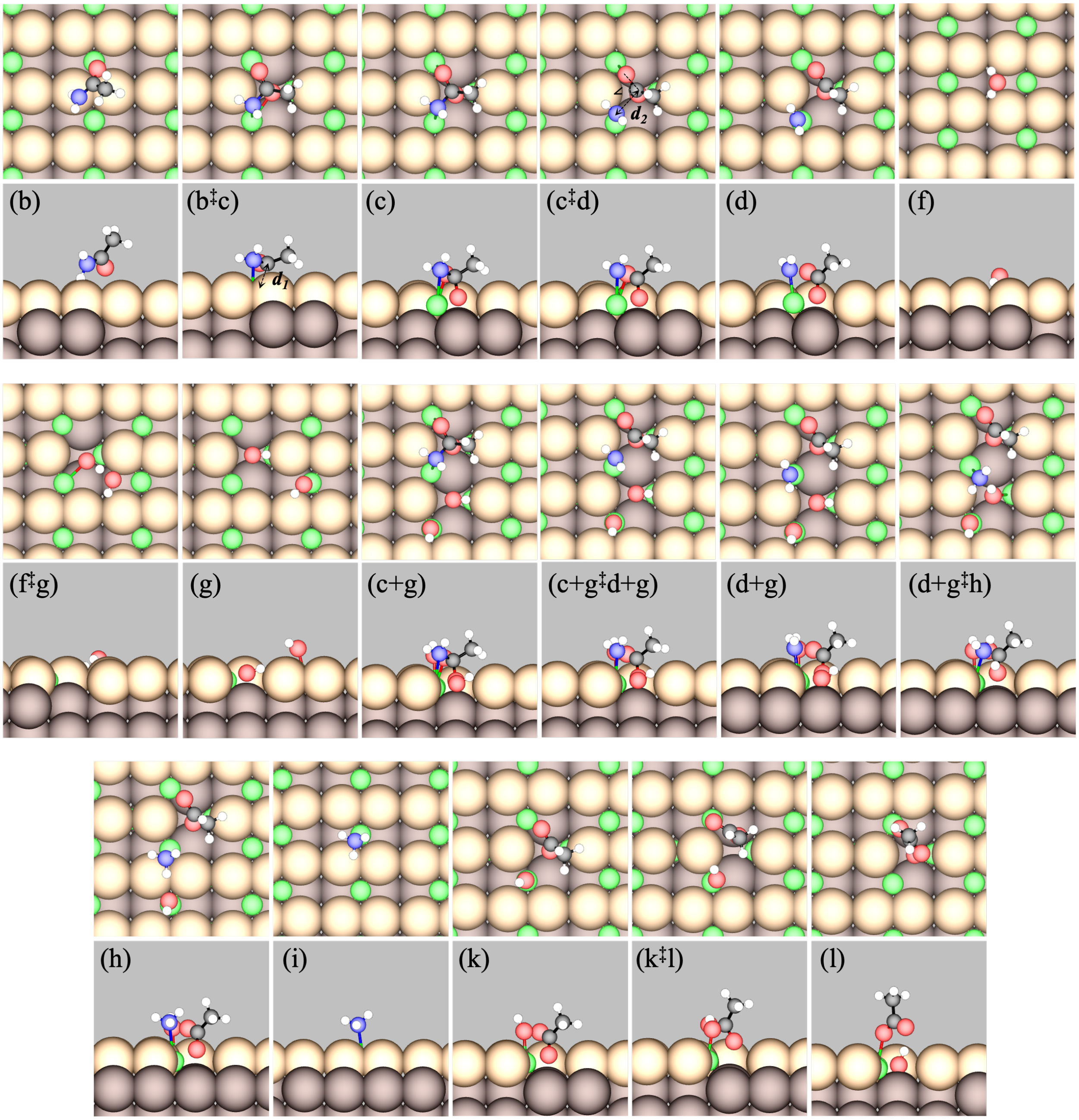

Figure S2. Top (upper panels) and side (lower panels) views of GGA-PW91 optimized stable intermediates and TSs (labeled ‡) of the elementary steps in deamidation and hydrolysis of acetamide on stoichiometric $CeO_2(110)$. Labels correspond to those in Table 2. The states shown are: (b) η^1 acetamide, (b‡c) TS for conversion to TI, (c) TI, (c‡d) TS of C–N scission, (d) acetyl + NH_2, (f) H_2O, (f‡g) TS for dissociation of H_2O, (g) OH + H, (c+g) TI + OH + H, (c+g‡d+g) TS of C–N scission with co-adsorbed dissociated water, (d+g) acetyl + NH_2 + OH + H, (d+g‡h) TS for hydrogenation of NH_2, (h) acetyl + NH_3

+ OH, (i) NH_3, (k) acetyl + OH, ($k^{‡}l$) TS of OH attack, and (l) acetic acid. Color code: Green=Ce, light brown=surface O_{latt}, dark brown=subsurface O_{latt}, red=O in molecules, black=C, blue=N, and white=H. O_{latt} bonded to C or H atoms in the molecules are considered part of the molecules. Labeled bond distances and angle are d_1 = 1.98 Å, d_2 = 2.14 Å, and ∠OCN = 102.7°.

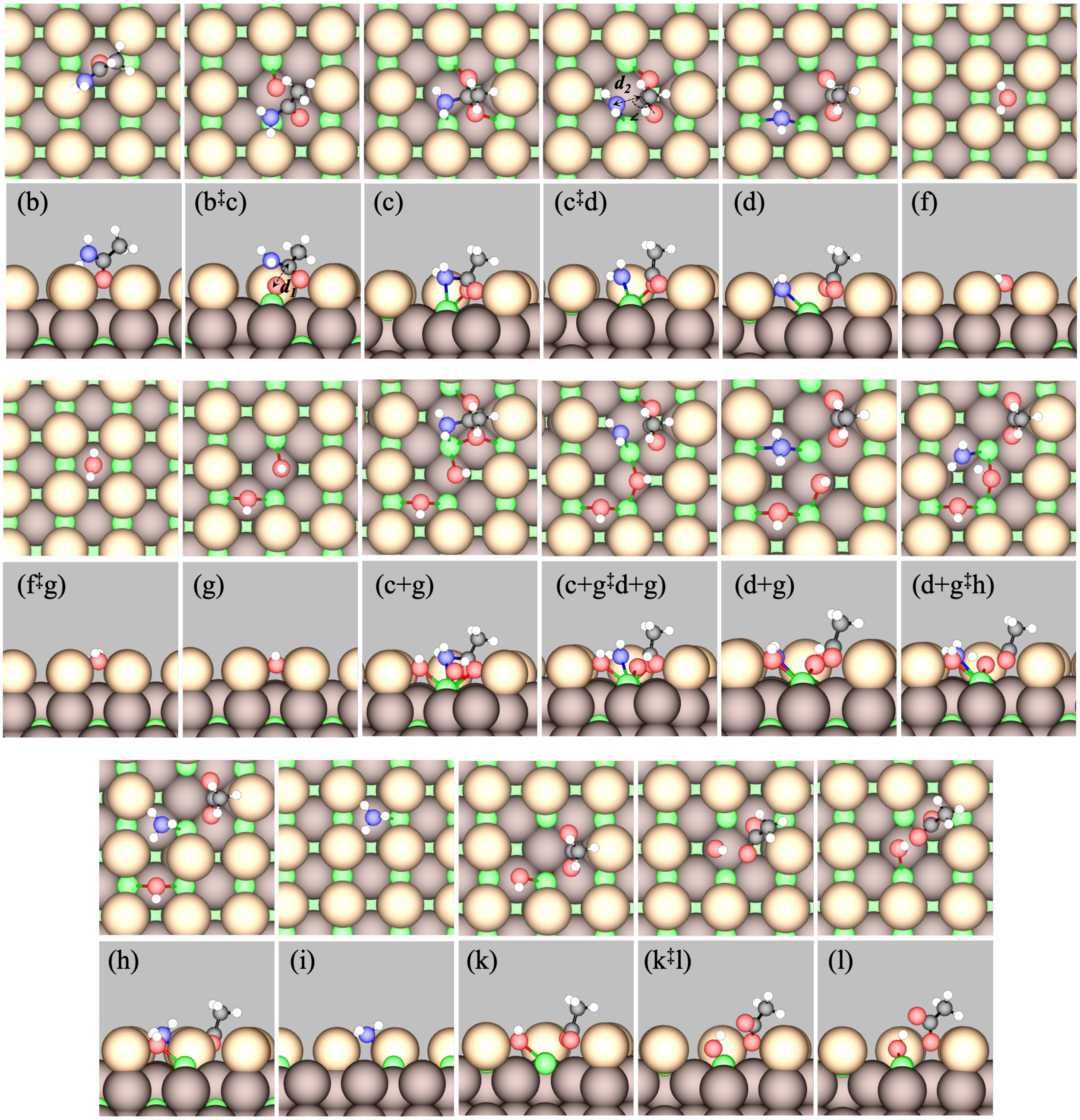

Figure S3. Top (upper panels) and side (lower panels) views of GGA-PW91 optimized stable intermediates and TSs (labeled ‡) of the elementary steps in deamidation and hydrolysis of acetamide on stoichiometric $CeO_2(100)$. Labels correspond to those in Table 2. The states shown are: (b) η^1 acetamide, (b‡c) TS for conversion to TI, (c) TI, (c‡d) TS of C–N scission, (d) acetyl + NH_2, (f) H_2O, (f‡g) TS for dissociation of H_2O, (g) OH + H, (c+g) TI + OH + H, (c+g‡d+g) TS of C–N scission with co-adsorbed dissociated water, (d+g) acetyl + NH_2 + OH + H, (d+g‡h) TS for hydrogenation of NH_2, (h) acetyl + NH_3

+ OH, (i) NH_3, (k) acetyl + OH, (k‡l) TS of OH attack, and (l) acetic acid. Color code: Green=Ce, light brown=surface O_{latt}, dark brown=subsurface O_{latt}, red=O in molecules, black=C, blue=N, and white=H. O_{latt} bonded to C or H atoms in the molecules are considered part of the molecules. Labeled bond distances and angle are d_1 = 1.90 Å, d_2 = 2.06 Å, and ∠OCN = 95.2°.

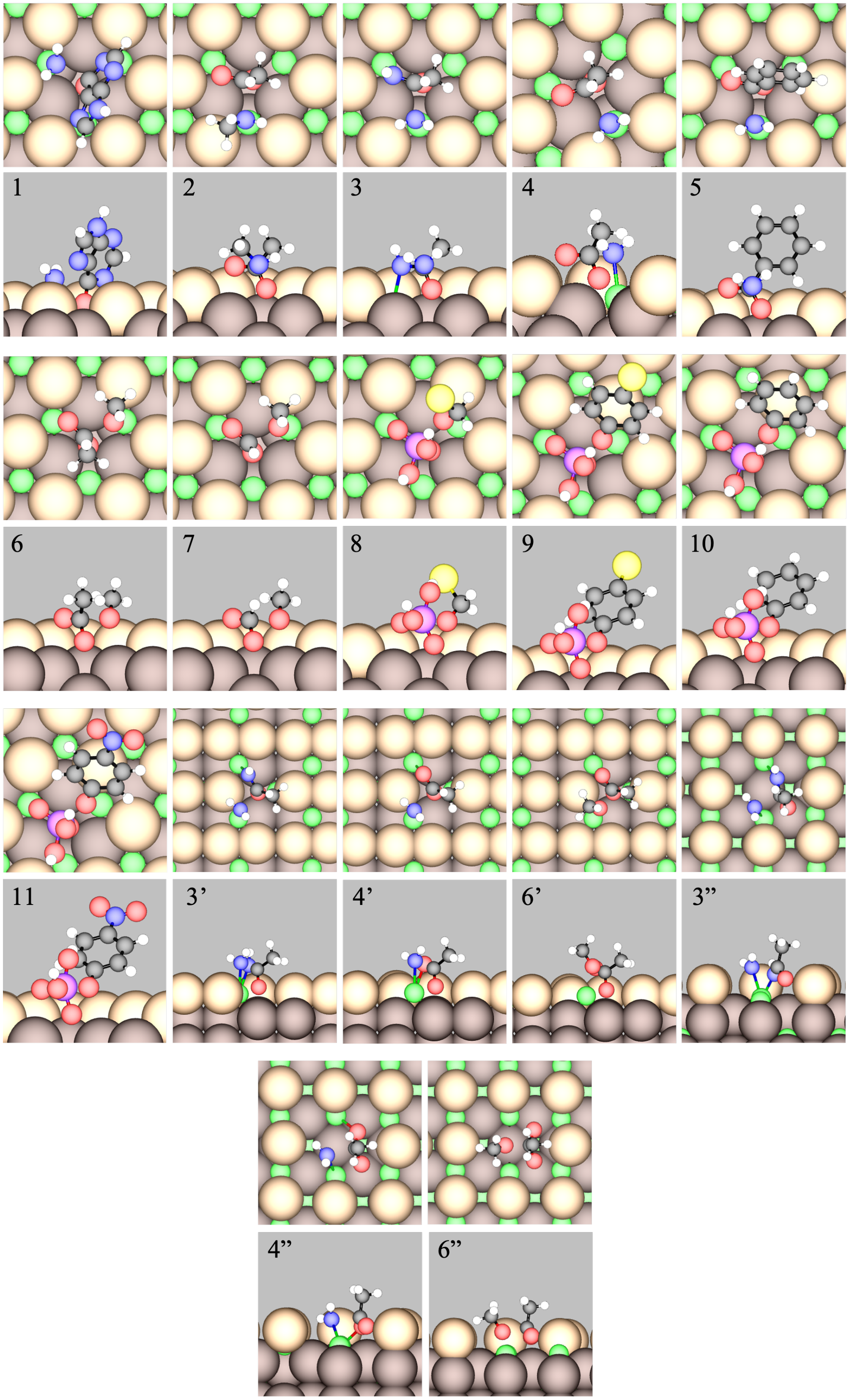
1
2
3
4
5
6
7
8
9
10
11
3’
4’
6’
3”
4”
6”

Figure S4. Top (upper panels) and side (lower panels) views of GGA-PW91 optimized TS of X–Z ester bond scission in GEs on the three low-index CeO_2 facets. Numberal labels correspond to those in Figure 8. The compounds included are: 1 adenine, 2 N-methylacetamide, 3 acetamidine, 4 acetamide, 5 benzamide, 6 methyl acetate, 7 methyl formate, 8 chloromethyl phosphate, 9 para-chlorophenyl phosphate, 10 phenyl phosphate, and 11 para-nitrophenyl phosphate. Unmarked numbers indicate results on (111), ' indicate results on (110), and " indicate results on (100). Color code: Green = Ce, light brown = surface O_{latt}, dark brown = subsurface O_{latt}, red = O in molecules, black = C, blue = N, violet = P, yellow = Cl, and white = H. O_{latt} bonded to C or P atoms in the molecules are considered part of the molecules.

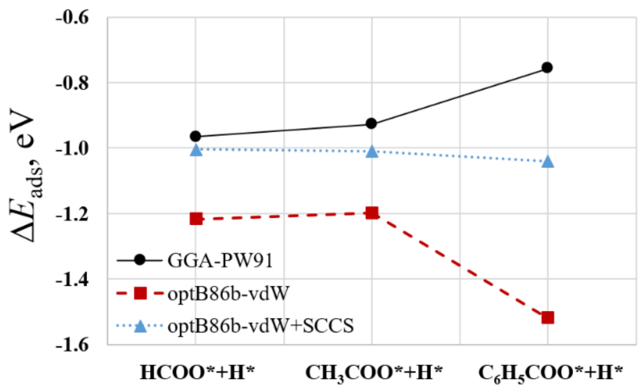

Figure S5. Comparison of the DFT-calculated minimum ΔE_{ads} of formic, acetic, and benzoic acids (as carboxylate + H) adsorbed on CeO_2(111), using GGA-PW91 (black circles, VASP), optB86b-vdW (red squares, QE), and optB86b-vdW+SCCS (blue triangles, QE).

Acetamide dehydration

Ceria can also catalyze the dehydration of acetamide to acetonitrile. The reaction would proceed via sequential loss of the amine hydrogens with concomitant surface reduction, in which $CH_3C(=O)NH$ displaces a hydrogenated O_{latt} so that the carbonyl O occupies a lattice position (similar to reduction of ceria by acetic acid mentioned above), followed by scission of the C–O bond to yield CH_3CN. The reaction energy profiles for acetamide dehydration on the three low-index facets of ceria are show in Figure S6 below.

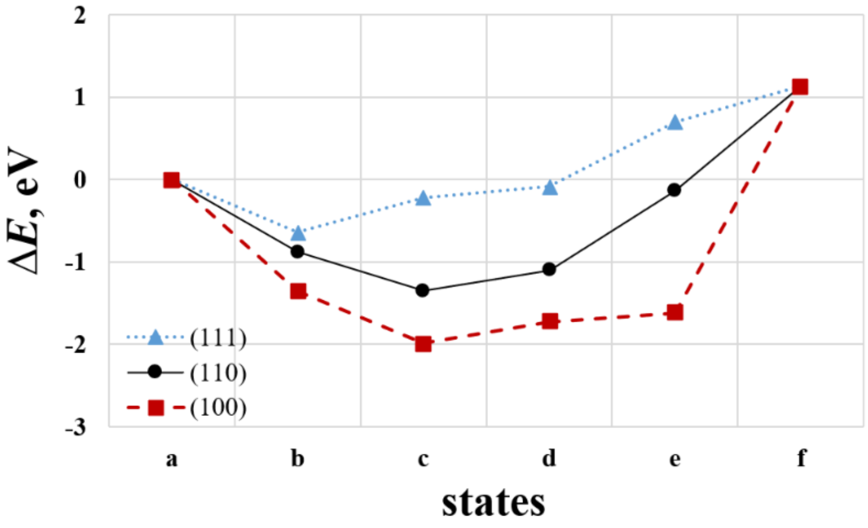

Figure S6. GGA-PW91 minimum-energy reaction energy profiles (stable states only) for dehydration in acetamide forming acetonitrile on the CeO_2(111), (110), and (100) facets. The reaction states are: (a) acetamide$_{(g)}$, (b) η^1 acetamide, (c) CH_3CONH + H, (d) CH_3CONH/V_O + OH, (e) CH_3CN + H_2O, and (f) acetonitrile$_{(g)}$ + $H_2O_{(g)}$. V_O corresponds to surface oxygen vacancy. Water in state € is molecular on (111) and dissociated (OH + H) on (110) and (100).

References

(1) Wang, X.; Zhang, K.; Zhao, W.; Zhang, Y.; Lan, Z.; Zhang, T.; Xiao, Y.; Zhang, Y.; Chang, H.; Jiang, L. Effect of ceria precursor on the physicochemical and catalytic properties of Mn–W/CeO_2 nanocatalysts for NH_3 SCR at low temperature. *Ind. Eng. Chem. Res.* **2017**, *56*, 14980–14994.

(2) Gao, R.; Zhang, D.; Maitarad, P.; Shi, L.; Rungrotmongkol, T.; Li, H.; Zhang, J.; Cao, W. Morphology-dependent properties of MnO_x/ZrO_2–CeO_2 nanostructures for the selective catalytic reduction of NO with NH_3. *J. Phys. Chem. C* **2013**, *117*, 10502–10511.

(3) Kamachi, T.; Siddiki, S. M. A. H.; Morita, Y.; Rashed, M. N.; Kon, K.; Toyao, T.; Shimizu, K.; Yoshizawa, K. Combined theoretical and experimental study on alcoholysis of amides on CeO_2 surface: A catalytic interplay between Lewis acid and base sites. *Catal. Today* **2018**, *303*, 256–262.

(4) Joshi, A.; Rammohan, A.; Jiang, Y.; Ogunwumi, S. Density functional theory (DFT) study of the interaction of ammonia with pure and tungsten–doped ceria. *J. Mol. Struct. THEOCHEM* **2009**, *912*, 73–81.

(5) Calaza, F. C.; Chen, T.-L.; Mullins, D. R.; Xu, Y.; Overbury, S. H. Reactivity and reaction intermediates for acetic acid adsorbed on CeO_2(111). *Catal. Today* **2015**, *253*, 65–76.

(6) Ren, Z.; Liu, N.; Chen, B.; Li, J.; Mei, D. Theoretical investigation of the structural stabilities of ceria surfaces and supported metal nanocluster in vapor and aqueous phases. *J. Phys. Chem. C* **2018**, *122*, 4828–4840.

(7) Molinari, M.; Parker, S. C.; Sayle, D. C.; Islam, M. S. Water adsorption and its effect on the stability of low index stoichiometric and reduced surfaces of ceria. *J. Phys. Chem. C* **2012**, *116*, 7073–7082.

(8) Fronzi, M.; Assadi, M. H. N.; Hanaor, D. A. H. Theoretical insights into the hydrophobicity of low index CeO_2 surfaces. *Appl. Surf. Sci.* **2019**, *478*, 68–74.

(9) Fernández-Torre, D.; Kośmider, K.; Carrasco, J.; Ganduglia-Pirovano, M. V.; Pérez, R. Insight into the adsorption of water on the clean CeO_2(111) surface with van der Waals and hybrid density

functionals. *J. Phys. Chem. C* **2012**, *116*, 13584–13593.

(10) Florián, J.; Warshel, A. Calculations of hydration entropies of hydrophobic, polar, and ionic solutes in the framework of the langevin dipoles solvation model. *J. Phys. Chem. B* **1999**, *103*, 10282–10288.
